# Extracellular Vesicle-Mimetic Nanoplatforms for Integrated Vaccination and Targeted Antibiotic Therapy against MRSA Bacteremia: A Wolf in Sheep’s Clothing

**DOI:** 10.34133/bmr.0411

**Published:** 2026-09-30

**Authors:** Yu-Kuo Chung, Cheng-Yu Lin, Yu-Ting Wang, Yen-Tzu Chang, Ahmed Alalaiwe, Huang-Ping Yu, Jia-You Fang

**Affiliations:** ^1^ Graduate Institute of Biomedical Sciences, Chang Gung University, Kweishan, Taoyuan, Taiwan.; ^2^ School of Pharmacy, College of Pharmacy, Taipei Medical University, Taipei, Taiwan.; ^3^ Pharmaceutics Laboratory, Graduate Institute of Natural Products, Chang Gung University, Kweishan, Taoyuan, Taiwan.; ^4^ Department of Radiation Oncology, The University of Texas MD Anderson Cancer Center, Houston, TX, USA.; ^5^ Department of Pharmaceutics, College of Pharmacy, Prince Sattam Bin Abdulaziz University, Al Kharj, Saudi Arabia.; ^6^ Department of Anesthesiology, Chang Gung Memorial Hospital at Linkou, Kweishan, Taoyuan, Taiwan.; ^7^ School of Medicine, College of Medicine, Chang Gung University, Kweishan, Taoyuan, Taiwan.; ^8^ Research Center for Food and Cosmetic Safety and Center for Drug Research and Development, Chang Gung University of Science and Technology, Kweishan, Taoyuan, Taiwan.

## Abstract

Methicillin-resistant *Staphylococcus aureus* (MRSA) remains a critical global health threat, necessitating innovative therapeutic strategies. Inspired by the multi-antigenic features and homotypic binding of MRSA extracellular vesicles (EVs), we aimed to develop a biomimetic nanoplatform cloaked with MRSA-derived EV, termed EV-NP, to integrate vaccine and antibiotic functionalities. EV-NP were efficiently internalized by macrophages and dendritic cells, promoting robust humoral and cellular immune responses, as evidenced by elevated immunoglobulin G titers and CD69 expression. When loaded with rifampicin (RF), the nanoplatform demonstrated homologous targeting to MRSA via EV-mediated adhesion, enhancing drug delivery to intracellular bacteria and biofilm. In a murine bacteremia model, EV-NP vaccination and RF-loaded EV-NP therapy each significantly reduced bacterial burden and proinflammatory cytokine expression, improving survival to 62 and 41 h, respectively. Sequential prophylaxis and therapy extended survival to 140 h. The EV-NP system also attenuated EV-associated hemolysis and showed no acute toxicity. This “wolf-in-sheep’s-clothing” strategy offers a versatile approach for both prevention and treatment of MRSA infection.

## Introduction

*Staphylococcus aureus* is a leading cause of sepsis and pneumonia, with methicillin-resistant strains (MRSA) presenting a major clinical challenge due to increased mortality and prolonged hospitalization [1]. Between 1990 and 2021, the mortality attributable to *S. aureus* antimicrobial resistance (AMR) rose from 13% to 19% [2], highlighting the urgent need for novel therapeutic strategies. Vaccines and antibiotics remain the mainstays for bacterial control, yet their efficacy against resistant pathogens remains limited. Recent advances in biomimetic nanoengineering offer promising avenues for both prophylactic and therapeutic applications against AMR bacteria [3]. By inheriting membrane proteins and antigens from parent cells, biomimetic nanoparticles (NPs) mimic cellular functions [4], enabling multi-antigenic presentation for immune activation and efficient lymphatic drainage for antigen delivery [5]. Moreover, their cell-derived homing capability enhances barrier penetration and site-specific delivery [6]. Extracellular vesicles (EVs) from bacteria can be harnessed to coat NPs, offering a versatile platform for vaccine development due to their immunogenicity, low cost, and ease of isolation [7].

Some EV-based vaccines, such as those targeting *Neisseria meningitidis*, have been clinically approved. However, challenges including toxicity, pathogenicity, stability, and scalability continue to hinder broader EV vaccine development [8]. For MRSA, genetic engineering is often required to attenuate virulence factors within EV to ensure safety [9]. An emerging strategy to overcome these limitations involves coating NPs with bacterial EV to generate bioinspired nanoformulations that are stable, uniform, and immunogenic [10]. Compared to native EV, EV-decorated NPs offer enhanced antigen-specific immune responses, improved drug loading, targeted delivery, and prolonged circulation. Meanwhile, the pipeline for novel antibiotics remains limited due to the high cost and slow development pace. Nanocarrier systems present an alternative strategy. Integrating bacterial EV with NPs enables homotypic targeting and efficient antimicrobial delivery against pathogenic bacteria. Despite this potential, there remains a lack of approved vaccines or antibiotic-loaded nanocarriers specifically for MRSA. Over the past decade, 9 *S. aureus* vaccine candidates have failed in clinical trials due to safety concerns and poor efficacy [11]. Combining vaccination with antibiotic therapy may offer synergistic protection. In this study, we developed a dual-functional nanoplatform by cloaking poly(lactic-co-glycolic) acid (PLGA) NPs with MRSA-derived EV (EV-NP), enabling both immune activation and antibiotic delivery. We planned to design a new nanoplatform against bacteremia. This modular nanoplatform can be readily adapted into 2 formulations: EV-NP without antibiotic loading for prophylactic vaccination and EV-RF NP with antibiotic loading for targeted treatment of established MRSA infection. Clinically, EV-NP without antibiotic encapsulation may serve as a prophylactic vaccine for individuals at high risk of MRSA exposure (e.g., the patients undergoing surgical procedures), inducing protective immunity before the onset of bacteremia. If infection subsequently occurs, the antibiotic-loaded EV-NP (EV-RF NP) can be administered as a targeted therapeutic strategy to eradicate circulating and intracellular MRSA, thereby producing a complementary prevention-to-treatment approach. This “wolf-in-sheep’s-clothing” strategy leverages bacterial components to outsmart the pathogen.

PLGA was chosen as the NP core for its biocompatibility and tunable properties. RF, a frontline antibiotic known to reduce *S. aureus* bacteremia recurrence [12], was incorporated as the model drug. We employed a bacteremia-induced sepsis model to evaluate both the immunogenic and therapeutic performance of the nanoplatform. *S. aureus* is the primary pathogen in bacteremia, with mortality rates of 15% to 30%, increasing to 20% to 50% for MRSA-induced cases [13,14]. Despite its clinical severity, effective treatments for bacteremia-related sepsis remain limited. This study demonstrates the potential of MRSA EV-coated nanocarriers as a unified platform offering both prophylactic and therapeutic benefits against MRSA bacteremia.

## Materials and Methods

### MRSA EV isolation

MRSA strain ATCC33591 was obtained from the American Type Culture Collection. The bacteria were inoculated into tryptic soy broth (TSB; BD Biosciences) and cultured in a shaker at 37 °C overnight. Then, the culture medium was diluted 1:20 with TSB broth, and MRSA was cultured for another day. The bacteria were pelleted by centrifugation at 8,000*g* for 10 min. The supernatant was filtered by 0.45-μm polyethersulfone filter. The EVs were pelleted by ultracentrifugation at 150,000*g* for 2 h and then resuspended with phosphate-buffered saline (PBS). Protein amount was quantified by bicinchoninic acid (BCA) protein assay. The EVs were stored at −20 °C for further use

### Fabrication of EV-NP

The PLGA NPs were formulated using an emulsification–solvent evaporation technique. Fifty milligrams of hydroxyl-terminated PLGA (lactide:glycolide = 1:1) and 4 mg of RF were dissolved in 4 ml of dichloromethane and quickly loaded into a 12-ml water dissolving 2.5% polyvinyl alcohol (PVA). The polymer emulsion was produced immediately after emulsification by probe sonicator for 5 min. The emulsion was then dropwise to 50 ml of 0.3% PVA under magnetic stirring at 500 rpm overnight. After removing the organic solvent and washing by PBS for 3 times, the polymeric NPs were isolated at 10,000 rpm for 10 min and diluted in water (1 ml). To construct EV-NP, EVs were mixed with NP at a 1:10 membrane protein-to-polymer ration and sonicated for 5 min.

### Characterization of the nanocarriers

The mean size, polydispersity index (PDI), and zeta potential were measured by Nano ZS90 analyzer (Malvern). The morphology of the NPs was visualized using transmission electron microscopy (TEM; Hitachi HT7800) and scanning electron microscopy (SEM; Hitachi SU8220). Verification of EV coating was performed using sodium dodecyl sulfate–polyacrylamide gel electrophoresis (SDS-PAGE). The nanoformulations in buffer were run on an SDS-PAGE in tris–glycine gel using an electrophoresis system at 120 V for 45 min. The total protein content of EV-NP was quantified using BCA assay. The resulting protein concentrations were used to normalize EV dosing. For stability assay, the nanoformulations were stored in PBS at 4 °C for 1 week to estimate size and surface charge.

### RF encapsulation percentage in nanocarriers

The RF-loaded NPs (RF NP and EV-RF NP) were dissolved in an equal volume of acetonitrile to completely disrupt PLGA matrix. The concentration of RF inside NPs was quantified using high-performance liquid chromatography (HPLC) equipped with a C18 column (LiChrospher, Merck). The mobile phase consisted of water and acetonitrile (3:7) adjusted to pH 2. RF was detected at a wavelength of 254 nm. The encapsulation efficiency was calculated based on the RF amount recovered from the NPs relative to the initial free RF.

### In vitro RF release from nanocarriers

RF release from RF NP and EV-RF NP was performed using the sample-and-separate method. The NP samples were added into PBS (1:4 dilution) and incubated with a magnetic stirrer in test tube at 37 °C with a rotational speed of 140 rpm. The samples were withdrawn at predetermined time intervals, followed by centrifugation at 10,000 rpm for 10 min. The supernatants were collected, and the concentration of released RF was quantified by HPLC.

### Proteomic profiles of EV and EV-NP

EV and EV-NP were lysed in protein extraction buffer containing 8 M urea, 50 mM ammonium bicarbonate, and protease inhibitor cocktail, followed by sonication on ice to disrupt vesicular membrane and release EV-associated proteins. The protein extracts from EV and EV-NP were diluted in ammonium bicarbonate and reduced with 5 mM dithiothreitol at 60 °C for 45 min, followed by alkylation with 10 mM iodoacetamide for 30 min. The samples were digested with sequencing-grade modified trypsin at 37 °C for 16 h. The resulting peptides were diluted with 0.1% formic acid and loaded into a reverse-phase Zorbax 300SB column (Agilent). Full-scan mass analysis was conducted on an Orbitrap mass analyzer (Thermo Fisher). Protein identification was carried out using Proteome Discoverer version 2.3 (Thermo Fisher). Mass/mass spectra were secured against the National Center for Biotechnology Information (NCBI) database using Mascot search engine version 2.5 (Matrix Science).

### Nanocarrier uptake by macrophages

Nanoformulations were labeled with rhodamine 800 (R800) (0.02%, Tokyo Chemical Industry) by vortex for 5 min to assess cell internalization using flow cytometry and confocal microscopy. The differentiation of THP-1 cell line to macrophage-like cells was performed before experiment [15]. THP-1 (5 × 10^5^ cells/ml) was exposed to the nanoformulations at 37 °C for 1 h. Subsequently the cells were washed with PBS 3 times and fixed with 4% formaldehyde before flow cytometry assay. The fluorescence was measured using an Attune NxT flow cytometer. THP-1 was stained with Hoechst 33342 (blue, Invitrogen) and LysoTracker (green, Invitrogen) to observe nuclei and lysosomes, respectively. The cells were photographed with a Leica TCS SP8X confocal microscope.

### Nanocarrier uptake by dendritic cells

Cellular uptake of the nanoformulations by dendritic cells was examined using DC2.4 cell line. EV and EV-NP were labeled with 1,1'-dioctadecyl-3,3,3′,3′-tetramethylindocarbocyanine perchlorate (DiI, 1 μg/ml, AAT Bioquest) by vortex for 5 min to enable fluorescence tracking. DC2.4 (5 × 10^5^ cells/ml) was incubated with EV or EV-NP at a final protein concentration of 5 μg/ml at 37 °C for 0.5, 1, and 2 h. After washing for 3 times, DiI fluorescence was examined by flow cytometry and confocal microscopy.

### Cell viability assay

The cell viability treated by the nanoformulations was analyzed using cell counting kit-8 (CCK-8). THP-1 and DC2.4 were cultured in RPMI 1640 with a cell density of 8 × 10^3^ cells/well at 37 °C for 24 h. Fibroblasts (Hs68) were cultured in Dulbecco’s modified Eagle’s medium (DMEM) with a density of 5 × 10^3^ cells/well. The EV and nanoformulations at protein concentrations of 0 to 1,000 ng/ml were added into the wells and incubated for 24 h. Then, the 5% CCK-8 reagent (Dojindo Laboratories) in culture medium (100 μl) was added into the wells, followed by incubation at 37 °C for 1 h. The absorbance was measured by a multiplate reader at 550 nm.

### Hemolysis

The blood samples were obtained from the healthy donors by venipuncture according to the protocol approved by the Institutional Review Board of Chang Gung Memorial Hospital. Erythrocytes were separated by centrifugation at 2,000*g* for 5 min and washed 3 times with normal saline. The erythrocytes were then resuspended in normal saline (2 ml). The nanoformulations (0.25 ml) were added to the suspension and incubated at 37 °C under magnetic stirring for 1 h. The samples were centrifuged at 2,000*g* for 5 min, and the absorbance of the supernatant was measured at 571 nm to determine hemolysis. Complete hemolysis induced by water was defined as 100%.

### Macrophage and dendritic cell activation

THP-1 and DC2.4 were seeded overnight into 24-well plates at 5 × 10^5^ and 1 × 10^6^ cells/well, respectively. After treatment with EV and nanoformulations for 24 h, the supernatant was collected. The cytokine level was analyzed using an enzyme-linked immunosorbent assay (ELISA) according to the manufacturer’s instructions (BioLegend). For the dendritic cell maturation test, adherent cells were collected using a cell scraper and pelleted by centrifugation at 700*g* for 5 min. The cell pellets were incubated with a staining cocktail containing fluorescein isothiocyanate (FITC)-conjugated anti-mouse CD40 (clone 3/23, BioLegend) and Alexa Fluor 647-conjugated anti-mouse CD86 (clone GL-1, BioLegend) for 30 min at 4 °C. After incubation, DC2.4 was washed and resuspended with 2 mM EDTA and 1% albumin for flow cytometry analysis.

### Animals

The female Balb/c mice (6 to 10 weeks old) were purchased from the National Laboratory Animal Center in Taiwan. All experimental procedures were performed in strict accordance with the guidelines approved by the Institutional Animal Care and Use Committee at Chang Gung University.

### In vivo IgG titer determination

EV and EV-NP were administered to mice via intramuscular injection on days 0, 7, and 14 at a protein dose of 0.5 μg. The reason of using intramuscular injection was the mimicry of the delivery route of most vaccines. The serum from each animal was sampled on days 0, 7, 14, and 21. Anti-MRSA titer was determined by ELISA. EVs as the captured antibody were dissolved in ELISA coating buffer on 96-well plates at 0.5 μg/well at 4 °C. The plates were then blocked at room temperature for 1 h with blocking buffer, incubated with serially diluted serum samples for 24 h, and probed with a horseradish peroxidase-conjugated anti-mouse immunoglobulin G (IgG) (BioLegend) for additional 2 h. The plates were developed with 3,3′,5,5′-tetramethylbenzidine substrate, and the reaction was stopped with 1 N HCl. The absorbance was read at 450 nm using a multiplate reader.

### In vivo T cell activation

The nanoformulations were administered intramuscularly into the thigh at a dose corresponding to 0.5 mg of PLGA. Spleens were excised 48 h after vaccination, mechanically dissociated, and processed into single-cell suspension. The cells were stained with fluorescently labeled antibodies against CD3 and CD4 to recognize T cell subsets, and CD69 was used as an activation marker. Samples were analyzed by a flow cytometer to quantify T cell activation following NP immunization.

### Time–growth curve assay and minimum inhibitory concentration

MRSA was diluted with TSB to achieve OD_600_ = 0.01. The MRSA population was treated with several dilutions of free and nanoparticulate RF. The absorbance of the bacterial solution was estimated by a multiplate reader at 600 nm within 24 h. Minimum inhibitory concentration (MIC) was defined as the RF concentration at which the absorbance was <0.1 [16].

### Intracellular MRSA infection

THP-1 and mouse neutrophils (5 × 10^5^ cells/ml) were infected with MRSA at 5 × 10^7^ colony-forming units (CFUs)/ml for 1 h. After infection, the supernatant was removed, and gentamicin at 200 μg/ml was added for 15 min to eradicate extracellular MRSA. Gentamicin solution was then aspirated, and the cells were washed with PBS to remove the remaining extracellular MRSA. The infected cells were subsequently treated with free and nanoparticulate RF for 1 h. The cells were lysed with 1% Triton X-100, and intracellular MRSA was quantified by plating serial dilutions of the lysates onto agar plates and incubated at 37 °C for 24 h to detect CFU.

### MRSA biofilm assay

R800-labeled nanoformulations were used to assess NP penetration within MRSA biofilm. The biofilm was established in a glass-bottom culture dish for 24 h. After biofilm formation, the culture medium was removed and replaced with NPs suspended in TSB for a 24-h incubation. The viable bacteria were stained with SYTO9 (green) for 15 min. The penetration and spatial distribution of NPs (red) were visualized using confocal microscopy.

MRSA (OD_600_ = 0.1) was incubated using TSB with 1% glucose at 37 °C for 4 and 24 h in 96-well plates to establish the biofilm. Following the treatment of free and nanoparticulate RF (200 ng/ml) for 24 h, each well received 125 μl of 0.1% crystal violet solution and incubated for 10 min. The absorbance was measured at 555 nm in the multiplate reader. To calculate the number of live MRSA in the biofilm, the supernatant of the biofilm was discarded and the biofilm bacteria were resuspended with PBS. The growth of MRSA inside the biofilm was loaded onto agar plate to estimate CFU. The live MRSA in the biofilm was also observed with confocal microscopy after SYTO9 staining for 15 min. The structural morphology of the biofilm was examined using SEM.

### Bacterial adherence test

MRSA, *S. aureus*, *S. epidermidis*, *Escherichia coli*, and *Pseudomonas aeruginosa* were cultured in TSB supplemented with glucose and adjusted to an initial density of OD_600_ = 0.1, followed by incubation for 24 h to allow surface attachment. The NPs were added to the adherent bacterial layer and co-incubated for 2 h to assess the adherence behavior. The samples were washed 3 times with PBS to remove unbound NPs and subjected to SEM processing. The specimens were fixed with 2.5% glutaraldehyde, dehydrated through a graded ethanol series, subjected to drying, mounted on aluminum stub, and sputter-coated with gold. The NP adhesion on bacterial surface was visualized under SEM.

### In vivo MRSA bacteremia model

A MRSA bacteremia animal model was established to evaluate the protective effects of NP vaccination and RF-loaded nanocarrier treatment. Mice were randomly assigned to vaccine group (MRSA, EV, and EV-NP) or therapeutic drug treatment group (MRSA, RF, RF NP, EV-RF NP). For the vaccine arm, mice received 2 immunizations on days −14 and −7 relative to MRSA infection with PBS (control group), EV, or EV-NP at a protein dose of 0.5 μg per intramuscular injection. We aimed to use intramuscular route for simulating clinical delivery of most vaccines. On day 0, bacteremia was induced by intravenous injection of 100 μl MRSA suspension (1 × 10^8^ CFU/ml). For the RF treatment arm, mice were intravenously administered with the formulations with RF-equivalence dose of 1 μg after MRSA infection for 1 h. In this case of antibiotic therapy, we used intravenous route for mimicking the clinical delivery of antibiotics for treating the acute inflammatory diseases. At 24 h post-infection, mice were sacrificed and the peripheral organs were collected. Organs were homogenized in PBS, and serial dilutions of the homogenates were plated on agar to determine CFU. Additionally, blood samples and organ homogenates were subjected to ELISA for quantifying cytokine levels.

### In vivo biodistribution of the nanocarriers

The nanocarriers with or without EV (EV-NP and NP) were labeled with 1,1′-dioctadecyl-3,3,3′,3′-tetramethylindotricarbocyanine iodide (DiR iodide, 1 mg/ml, AAT Bioquest) to observe the biodistribution in vivo. Mice were anesthetized through intraperitoneal injection of Zoletil (25 mg/kg) and xylazine (6 mg/kg), followed by intravenous administration of the nanocarriers through the tail vein. The whole-body near-infrared imaging was detected for 60 min using a Pearl Impulse Imaging System (LI-COR). Then, the animals were euthanized, and the peripheral organs were collected and rinsed with saline. The near-infrared signal in the organs was visualized and quantified.

### In vivo survival rate

The mice were infected by intravenous injection of 100 μl of MRSA suspension (1 × 10^9^ CFU/ml). The experimental groups included MRSA only, RF, vancomycin, RF NP, EV-RF NP, EV-NP, and the combination of EV-NP vaccination followed by EV-RF NP treatment (EV-NP→EV-RF NP). Vaccination and therapeutic treatment were conducted following the same timing and procedure described in the MRSA biofilm assay section. Mice were monitored for survival over 10 d.

### The safety of the nanocarriers in healthy mice

The safety profiles of the nanoformulations were evaluated in healthy mice receiving EV-NP and/or EV-RF NP. The protocol for administering the nanoformulations was the same as that used in bacteremia model. All animals were monitored for general health, weigh change, and clinical signs. At 24 h after the final administration, the mice were sacrificed for collecting the blood samples for serum biochemistry analysis. The peripheral organs were also collected for histological examination.

### Statistical analysis

The data in the present study were employed as the mean and standard error of the mean (SEM). The significant difference between different groups was examined with the Kruskal–Wallis test, while the post hoc test was conducted with Dunn’s test. The significance was demonstrated by *P* < 0.05 (*), 0.01 (**), and 0.001 (***).

## Results

### Characterization of the nanocarriers

To construct the MRSA EV-coated nanoplatforms, PLGA NPs (146 nm) were synthesized via an emulsification–solvent evaporation method (Table 1) and subsequently fused with MRSA-derived EV through sonication (Fig. 1A). The native EV had an average size of 110 nm, consistent with previous reports [17]. Following EV cloaking, the particle size increased slightly to 169 nm, confirming successful membrane fusion. Drug loading with RF did not alter the size (Fig. 1B). While native EV showed a broader size distribution (PDI = 0.31), both EV-NP and EV-RF NP exhibited narrow dispersity (PDI = 0.06 and 0.03, respectively) (Table 1). Zeta potential analysis revealed a shift from −17 mV for bare PLGA NPs to −26 mV for EV-NP, comparable to EVs (−25 mV), further supporting surface coating (Fig. 1C). Protein analysis via BCA assay and SDS-PAGE confirmed the presence of EV-associated proteins on EV-NP, with preserved protein profiles after coating (Fig. 1D and E). The encapsulation percentage of RF in RF NP and EV-RF NP was 1.27% and 1.20%, respectively. To ensure the complete RF loading in the NPs for examining the biological benefit of nanoparticulate drug over free RF, we removed the unencapsulated RF molecules before the test. The nanocarriers with full RF entrapment were reconstituted with water to obtain the determined concentration. The drug release from RF NP and EV-RF NP achieved 80% in the initial 6 h (Fig. S1). After this time, few RF molecules were further released from the nanocarriers. The NPs maintained about 20% RF inside the matrix for 48 h. Both nanoformulations exhibited a comparable RF release.

**Table 1.** Physicochemical properties of the nanoparticles. Data are presented as mean ± SEM (*n* = 3).

Formulation	Size (nm)	PDI	Zeta potential (mV)
PLGA	145.56 ± 4.13	0.08 ± 0.03	−17.16 ± 0.31
EV	110.33 ± 21.23	0.31 ± 0.09	−25.20 ± 1.22
EV-NP	168.83 ± 6.21	0.06 ± 0.02	−25.56 ± 0.47
EV-RF NP	169.63 ± 3.38	0.03 ± 0.01	−24.67 ± 0.51

**Fig. 1.**
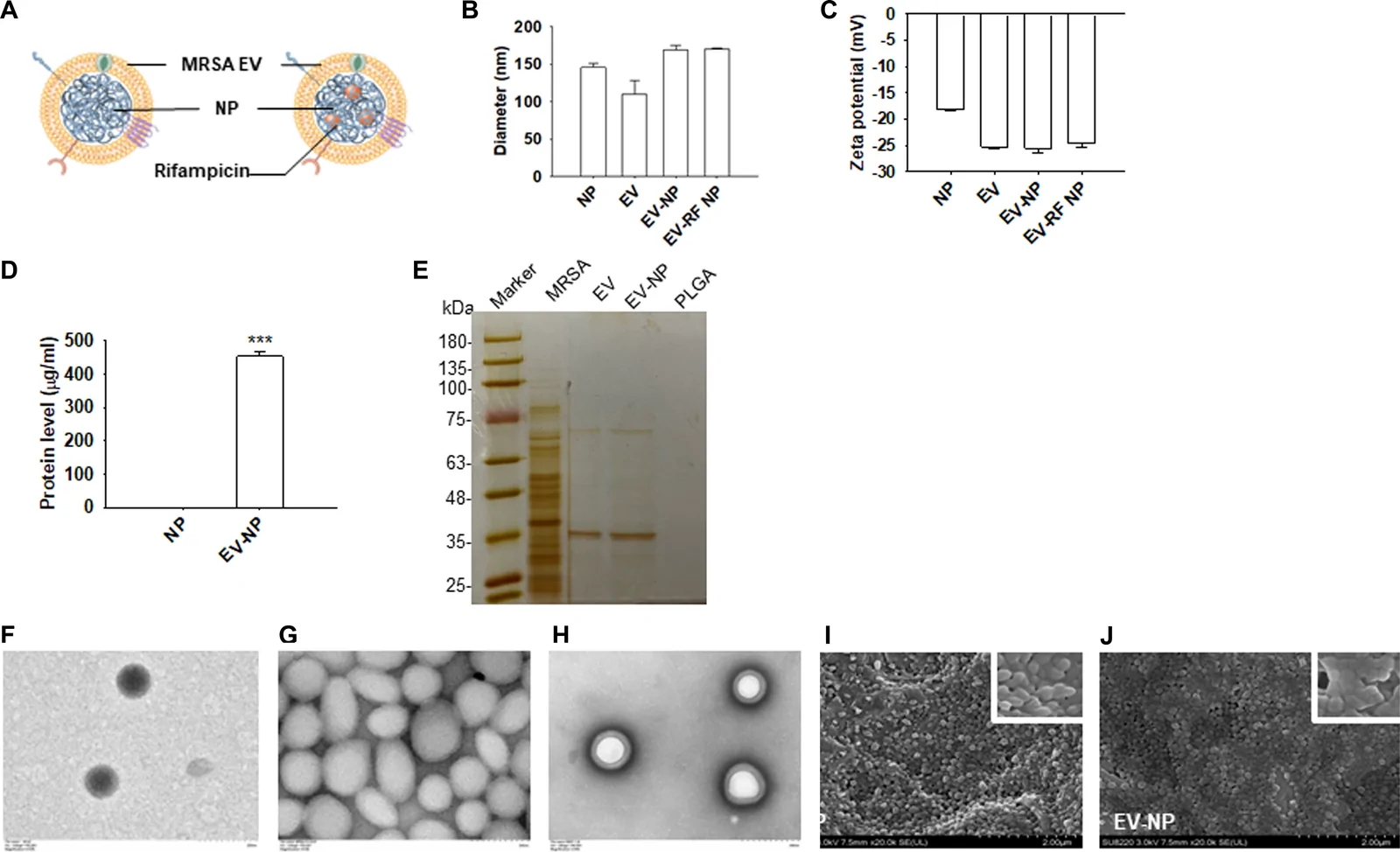
Morphology and EV coating of rifampicin-loaded NPs. (A) Schematic illustration of the NP systems, including EV-NP and EV-RF NP. (B and C) Hydrodynamic diameter (B) and zeta potential (C) of bare PLGA NPs, native EV, EV-NP, and EV-RF NP. (D) Quantification of protein content confirming successful EV membrane coating on NPs. Data are presented as mean ± SEM (*n* = 3). Compared with the NP group: ***P < 0.001. (E) Silver staining showing protein profiles of EV and EV-NP. (F to H) TEM images of NP (F), EV (G), and EV-NP (H), demonstrating the presence of a core–shell structure after EV coating. (I and J) SEM images of NP (I) and EV-NP (J) depicting morphological differences associated with EV membrane decoration.

Proteomic analysis confirmed similar protein profiles between EV and EV-NP, with the top 50 most abundant proteins listed in Fig. S2. In EV, lipase 2 and autolysin were the most dominant, alongside several ribosomal proteins and hemolysins. EV-NP preserved these key components, with autolysin and lipase 2 ranking highest. Notably, the presence of immunogenic proteins such as immunodominant staphylococcal antigen B (IsaB), hemolysins, and leukocidin-like proteins (LUKLs) in EV-NP suggests potential for immune activation.

TEM imaging showed that bare PLGA NPs were spherical (130 nm) (Fig. 1F), while isolated EV exhibited smooth spherical or ovoid morphology (Fig. 1G). EV-NP displayed a characteristic core–shell structure with a 20- to 30-nm-thick EV membrane, confirming successful cloaking (Fig. 1H). TEM-estimated diameters for EV-NP ranged from 120 to 150 nm, slightly smaller than Zetasizer results. SEM analysis further revealed smooth surfaces and uniform size distributions for both NP and EV-NP (Fig. 1I and J).

### In vitro cell uptake and toxicity assay

Before conducting cell-based experiments, we first evaluated the storage stability of the nanoformulations. Over 7 d at 4 °C, EV-camouflaged NPs showed no significant changes in size or zeta potential (Fig. 2A and B), indicating acceptable stability. All formulations were used within 7 d of preparation.

**Fig. 2.**
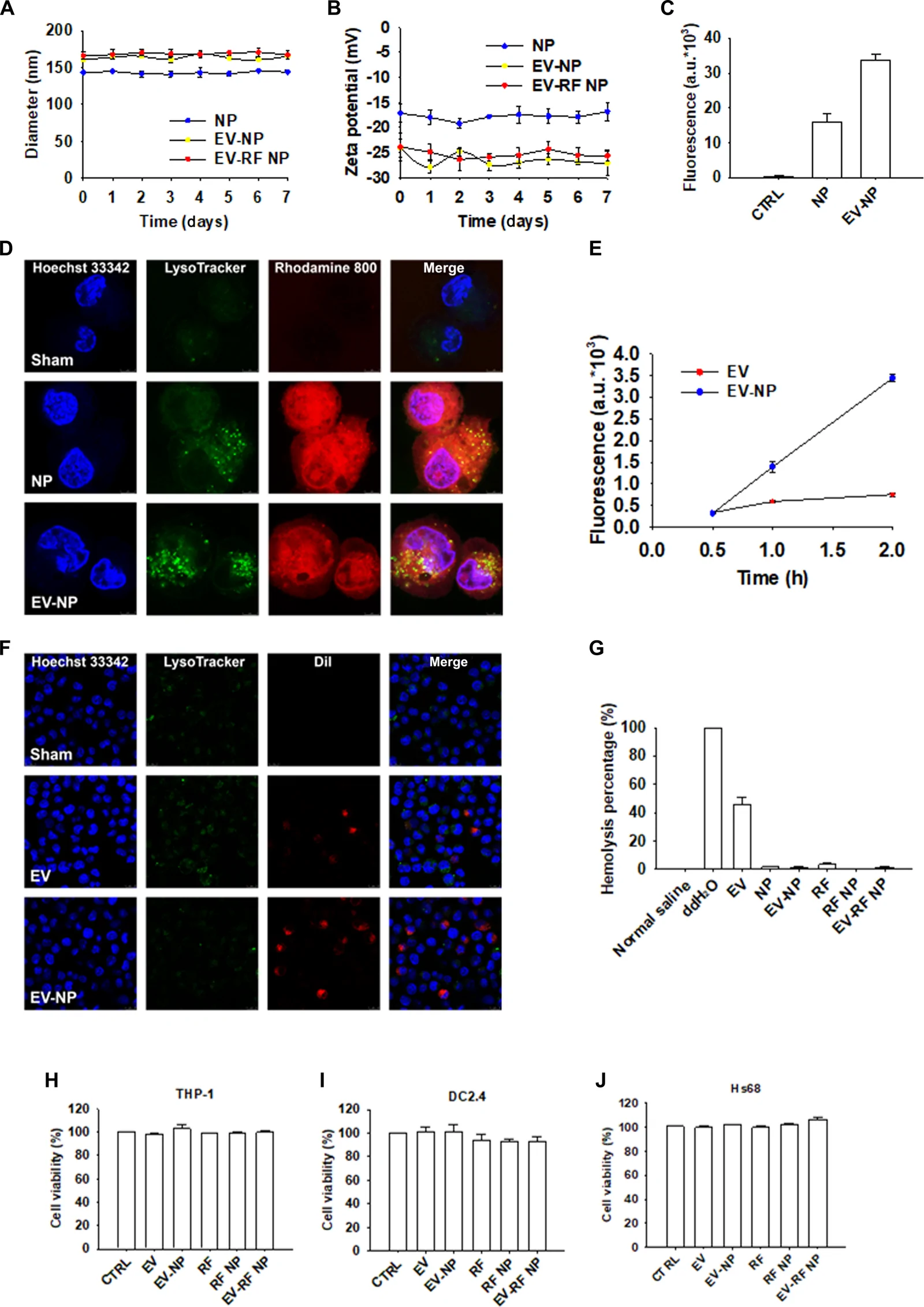
The physicochemical stability, cellular uptake, and biocompatibility of nanocarriers. (A and B) Stability of NPs in PBS, including changes in hydrodynamic diameter (A) and zeta potential (B). Data are presented as mean ± SEM (*n* = 3). (C) Flow cytometry analysis of dye-labeled NP and EV-NP uptake by THP-1 cells. (D) Confocal fluorescence images showing intracellular localization of NP and EV-NP in THP-1 cells after 1 h of incubation. (E) Time-dependent uptake of dye-labeled native EV and EV-NP by DC2.4 dendritic cells. Data are presented as mean ± SEM (*n* = 3). (F) Confocal fluorescence images showing NP and EV-NP uptake in DC2.4 cells after 2 h of incubation. (G) Hemolysis assay of the nanocarriers in human erythrocytes. (H to J) Cytotoxicity of the formulations toward THP-1 (H), DC2.4 (I), and Hs68 (J) cells, measured using the CCK-8 assay. Data are presented as mean ± SEM (*n* = 3).

We next assessed NP uptake by macrophages and dendritic cells. Compared to dye-labeled PLGA NPs, EV-NP exhibited significantly higher internalization by macrophages, as detected by flow cytometry (Fig. S3A), with approximately 2-fold greater fluorescence intensity (Fig. 2C). Confocal imaging showed bright red fluorescence localized in the cytoplasm and perinuclear regions, appearing as punctate dots (Fig. 2D). NPs also colocalized with lysosomal compartments stained with LysoTracker.

Dendritic cell uptake was also examined. Given that dendritic cells are key antigen-presenting cells (APCs), EVs were included for comparison. Flow cytometry revealed higher uptake of EV-NP compared to EV (Fig. 2E), with time-dependent increases in internalization (Fig. S3B). Confocal microscopy further confirmed more efficient uptake of EV-NP than EV after 2 h of incubation (Fig. 2F).

The in vitro toxicity of the nanoformulations was assessed via hemolysis and cell viability assays. EV alone induced significant hemolysis when incubated with erythrocytes (Fig. 2G). However, this effect was completely abolished upon NP surface coating, with all nanoformulations (with or without RF) showing no hemolytic activity. At an equivalent dose of 1,000 ng/ml, none of the tested treatments (EV, free RF, or nanoformulations) reduced the viability of macrophages or dendritic cells (Fig. 2H and I). Similarly, no cytotoxicity was observed in normal human fibroblasts (Hs68) (Fig. 2J). Across all cell types, NP-treated groups maintained >90% viability at RF concentrations ranging from 100 to 1,000 ng/ml (Fig. S4), indicating excellent biocompatibility.

### EV-coated nanocarriers enhance the activation in immune cells

Bacterial EVs are known to stimulate both innate and adaptive immune responses by activating macrophages, dendritic cells, T cells, and B cells. We first evaluated the immunostimulatory effects of the biomimetic NPs on APCs, focusing on cytokine production. Bare PLGA NPs had no effect on tumor necrosis factor-α (TNF-α) or interleukin-6 (IL-6) levels in macrophages (Fig. 3A and B). In contrast, EV significantly up-regulated both cytokines, and EV-NP elicited an even stronger response. EV-NP treatment resulted in a 45- and 47-fold increase in TNF-α and IL-6, respectively, compared to control. Both EV and EV-NP induced cytokine production in a dose-dependent manner (Fig. S5A to D).

**Fig. 3.**
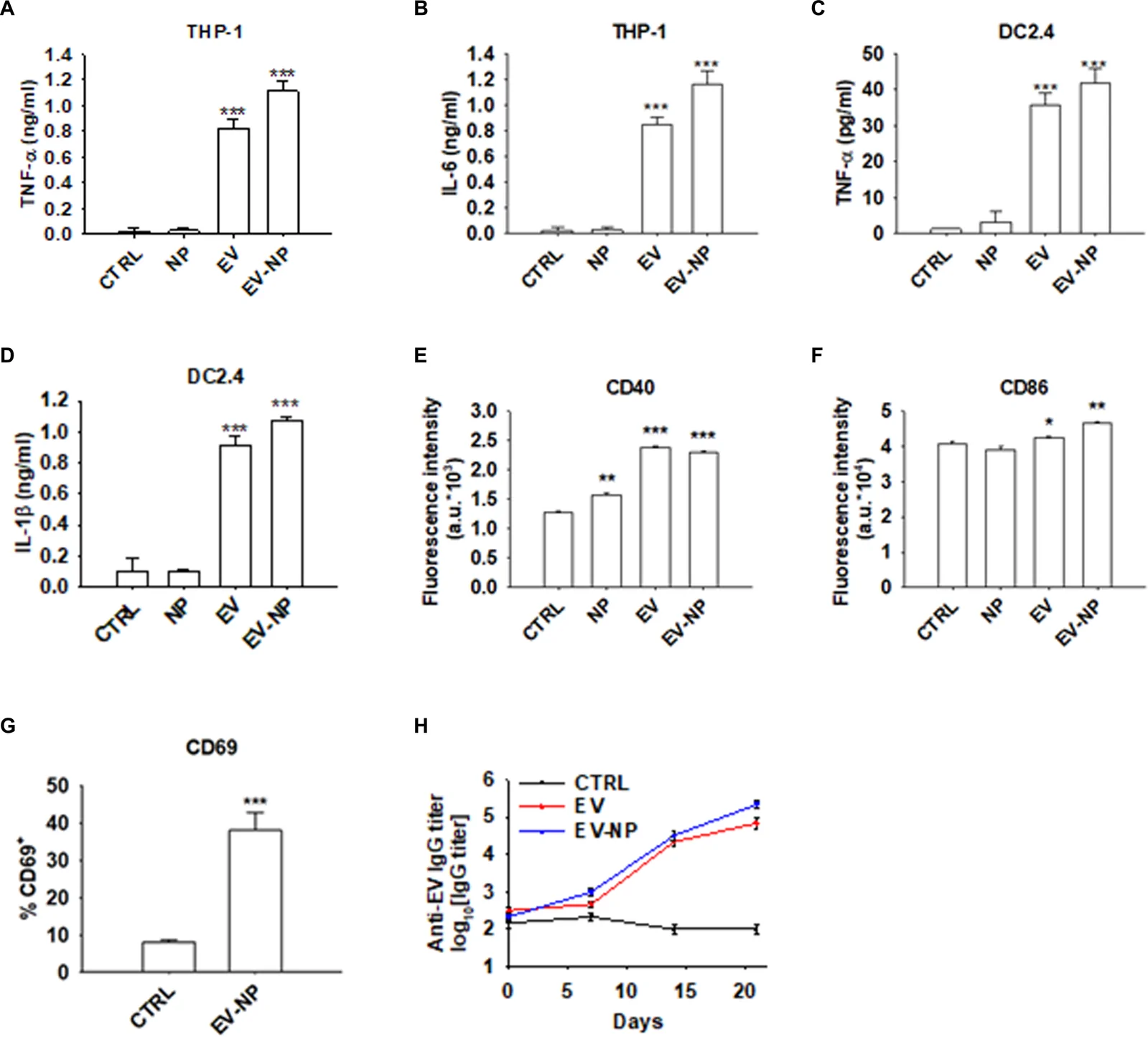
In vitro and in vivo immune stimulation. (A and B) Secretion of TNF-α (A) and IL-6 (B) from THP-1 cells after 24 h of incubation with EV-NP or control formulations. (C and D) Secretion of TNF-α (C) and IL-1β (D) from DC2.4 dendritic cells after 24 h of incubation with EV-NP or control formulations. (E and F) Dendritic cell activation following vaccination. Expression levels of the maturation markers CD40 (E) and CD86 (F) in dendritic cells after EV-NP vaccination. (G) Early T cell activation in vivo. Expression of the activation marker CD69 on splenic CD4^+^ T cells following EV-NP vaccination. (H) Adaptive immune responses induced by vaccination. Total anti-MRSA IgG titers in mouse serum on day 21 following immunization on days 0, 7, and 14 with native EV or EV-NP. Data are presented as mean ± SEM (*n* = 6). Compared with the control (CTRL) group: *P < 0.05, **P < 0.01, ***P < 0.001.

Similarly, EV and EV-NP activated dendritic cells, as shown by increased TNF-α and IL-1β levels (Fig. 3C and D and Fig. S5E to H), with EV-NP again exhibiting superior potency. To assess dendritic cell maturation, we measured surface markers CD40 and CD86. Flow cytometry revealed that both EV and EV-NP significantly up-regulated these markers, with EV-NP inducing a 2-fold higher CD40 expression compared to untreated controls (Fig. 3E and F).

To evaluate adaptive immune responses, we assessed T and B cell activation in vivo following vaccination. EV-NP significantly increased CD69 expression on splenic CD4^+^ T cells, indicating early T helper (Th) cell activation (Fig. 3G and Fig. S6). As CD4^+^ T cells play a central role in orchestrating adaptive immunity, this response suggests effective T cell priming. B cell activation was confirmed by increased serum IgG titers, as determined by ELISA (Fig. 3H). Both EV and EV-NP vaccination enhanced IgG titers in a time-dependent manner, with EV-NP preserving the immunogenic potential of native EVs.

### EV-coated nanocarriers eliminate planktonic and intracellular MRSA

To assess antibacterial efficacy, RF was encapsulated into the nanocarriers, with unbound drug removed to ensure accurate dosing. Free RF eliminated planktonic MRSA with a MIC of 100 to 200 ng/ml (Table S1), while RF NP and EV-RF NP showed enhanced potency, reducing the MIC to 50 to 100 ng/ml and 25 to 50 ng/ml, respectively. Time–kill assays showed that free RF at 25 and 50 ng/ml failed to sustain MRSA suppression beyond 10 h (Fig. 4A), whereas both RF NP and EV-RF NP fully eradicated bacteria within 24 h at doses ≥25 ng/ml (Fig. 4B and C). At 12.5 ng/ml, EV-RF NP exhibited the strongest inhibition, outperforming RF NP and free RF (Fig. 4D). For intracellular MRSA, free RF reduced bacterial load in macrophages by about 1 log, while RF NP and EV-RF NP achieved 1.5- and 2-log reductions, respectively (Fig. 4E). Similar trends were observed in neutrophils, where EV-RF NP demonstrated superior bacterial clearance (Fig. 4F).

**Fig. 4.**
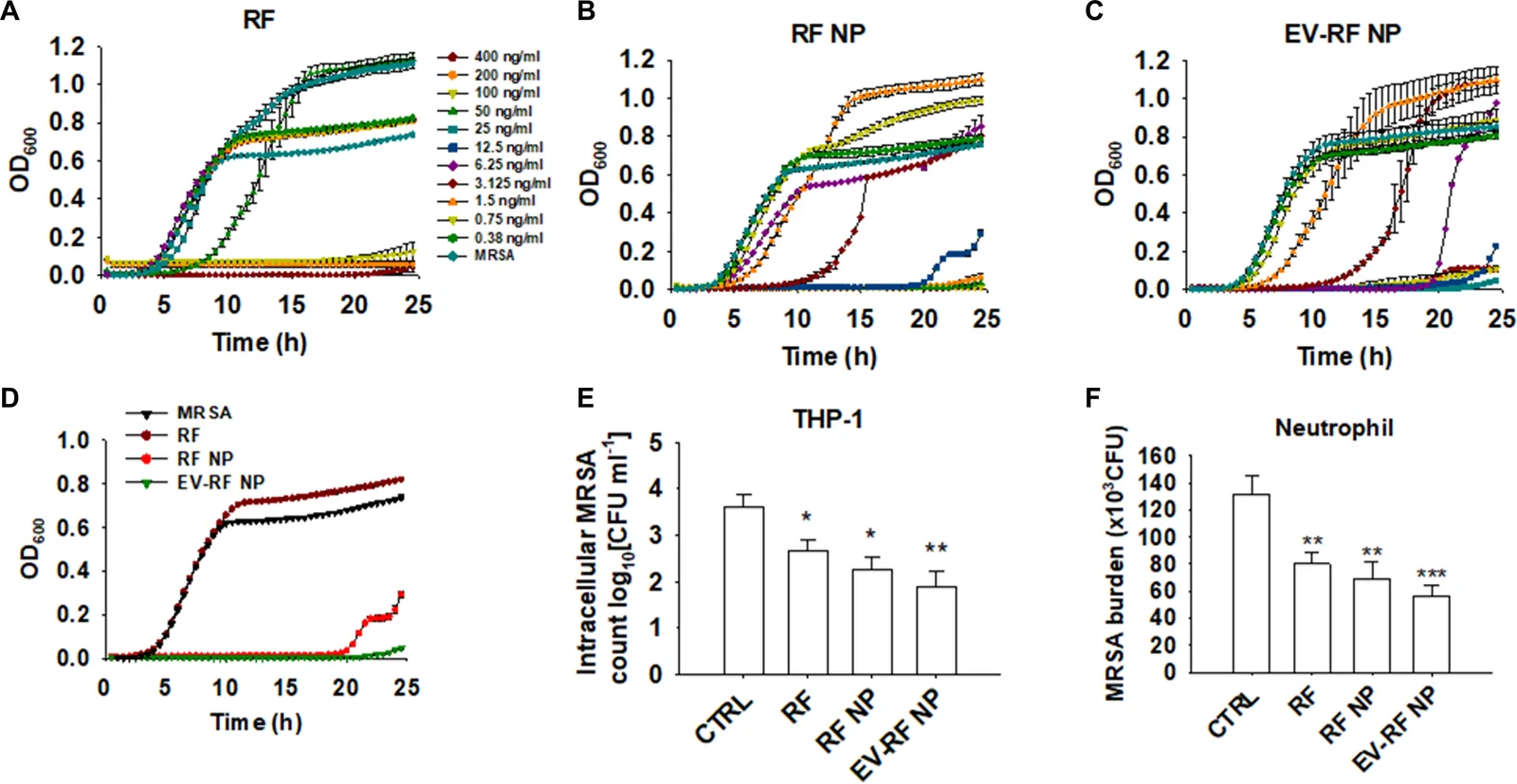
In vitro anti-MRSA activity of the nanoformulations. (A to C) Time–kill growth curves of MRSA treated with RF (A), RF NP (B), or EV-RF NP (C) at concentrations ranging from 400 to 0.038 ng/ml. Data are presented as mean ± SEM (*n* = 3). (D) Comparison of the growth inhibition of MRSA treated with RF, RF NP, or EV-RF NP at 12.5 ng/ml. (E and F) Intracellular antibacterial activity of RF, RF NP, and EV-RF NP after 1 h of treatment in THP-1 macrophage-like cells (E) and primary mouse neutrophils (F). Data are presented as mean ± SEM (*n* = 5). Compared with the control (CTRL) group: *P < 0.05, **P < 0.01, ***P < 0.001.

### EV-coated nanocarriers eliminate biofilm MRSA

MRSA biofilms pose a major barrier to antibiotic penetration. Three-dimensional confocal imaging revealed that R800-labeled NP and EV-NP penetrated deeply and uniformly throughout the biofilm matrix after 24 h (Fig. 5A and B). Both immature (4 h) and mature (24 h) biofilms, stained with crystal violet, showed significantly greater biomass disruption following treatment with nanocarriers compared to free RF, with EV-NP exhibiting the strongest effect (Fig. 5C and D). In immature biofilms, RF NP and EV-RF NP reduced viable MRSA by 1-log, while free RF showed no effect (Fig. 5E). In mature biofilms, EV-RF NP and RF NP achieved a 4-log reduction, whereas free RF only reduced MRSA by 2-log (Fig. 5F). Confocal microscopy of SYTO9-stained biofilms confirmed decreased green fluorescence after NP treatment, indicating bacterial elimination (Fig. 5G).

**Fig. 5.**
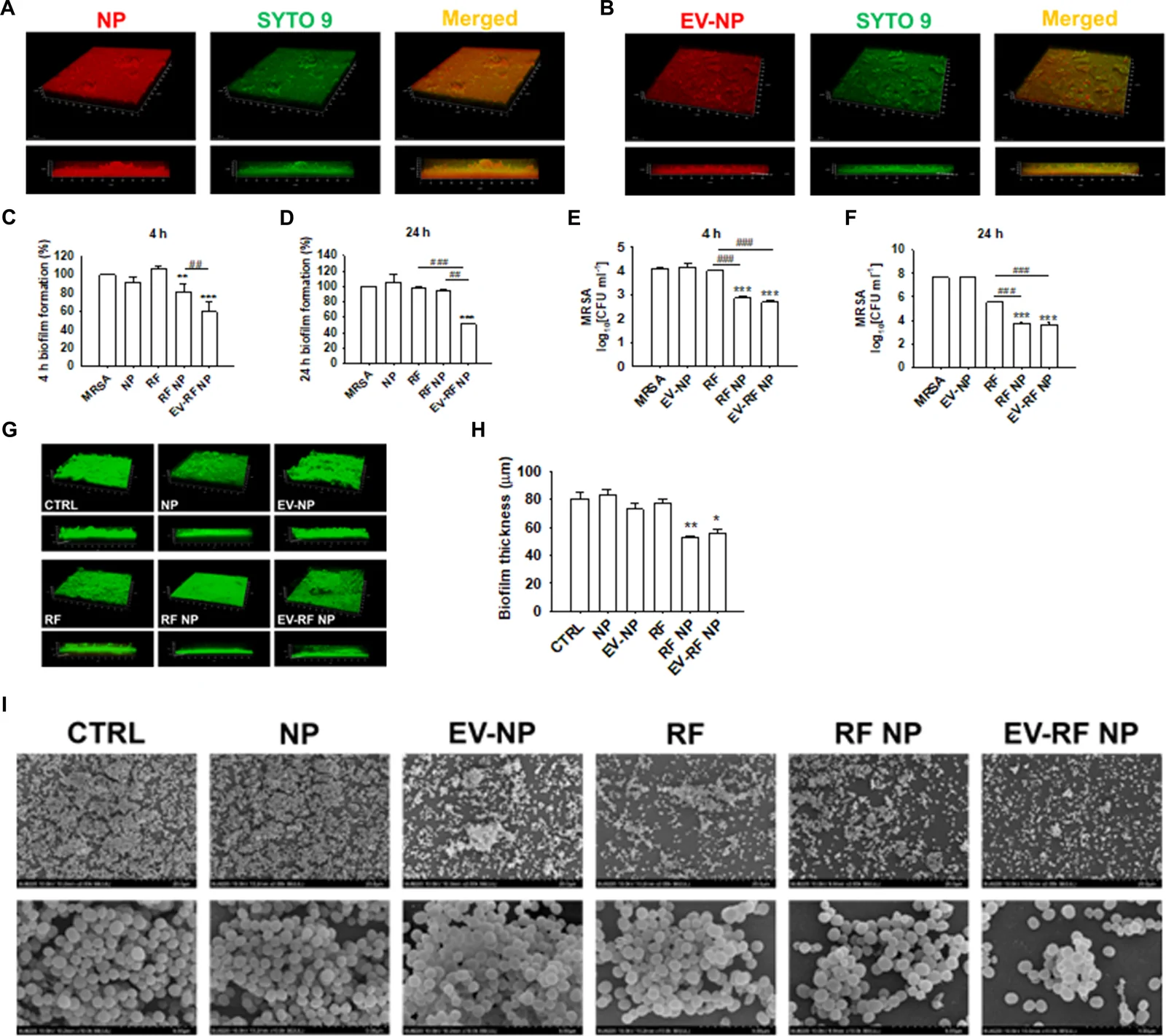
In vitro antibiofilm activity of the nanoformulations. (A and B) Penetration of NP (A) and EV-NP (B) into preformed MRSA biofilms observed by confocal laser scanning microscopy. Biofilm-embedded MRSA were stained with SYTO9 (green), and nanocarriers were labeled with R800 (red). (C to F) Biofilm inhibition quantified by crystal violet assay (C and D) and CFU enumeration (E and F) at the early-stage (4 h) and mature (24 h) biofilm. Data are presented as mean ± SEM (*n* = 3). (G and H) Structural disruption of biofilm following treatment. (G) Confocal laser scanning microscopy (CLSM) visualization of biofilm architecture stained with SYTO9 (green) and (H) quantification of biofilm thickness after 24 h of treatment. (I) SEM imaging of MRSA biofilms after treatment with different nanoformulations, showing morphological alterations and reduced bacterial density. Compared with the untreated MRSA group: *P < 0.05, **P < 0.01, ***P < 0.001. RF versus RF NP versus EV-RF NP: *P* < 0.05, ^##^*P* < 0.01, ^###^*P* < 0.001.

Biofilm thickness decreased from 81 nm to 53 nm and 56 nm after RF NP and EV-RF NP treatment, respectively, whereas free RF failed to reduce thickness despite reducing bacterial load (Fig. 5H). SEM imaging showed dense MRSA aggregates embedded in extracellular polymeric substances (EPSs) under control conditions. Treatment with RF formulations disrupted biofilm architecture, with signs of bacterial membrane damage. NPs without RF (NP and EV-NP) had negligible structural effects (Fig. 5I).

The bacterial binding capability of the nanoformulations was assessed by SEM. After 2-h incubation, EV-NP were visibly attached to MRSA surfaces, whereas bare NPs showed no such adherence (Fig. 6A). To assess binding specificity, other bacteria including *S. aureus*, *S. epidermidis*, *E. coli*, and *P. aeruginosa* were tested. Only MRSA exhibited EV-NP attachment, confirming homologous recognition (Fig. 6B). Time–kill assays further supported this specificity. EV-RF NP exhibited superior MRSA inhibition compared to RF NP (Fig. 6C), a difference not observed in other strains (Fig. 6D to G). Free RF eradicated *S. aureus* and *S. epidermidis* effectively (Fig. 6D and E). This anti-*S. aureus* and anti-*S. epidermidis* activity of free antibiotic was comparable to that of EV-RF NP. In Gram-negative bacteria, free RF outperformed nanocarriers in growth inhibition (Fig. 6F and G).

**Fig. 6.**
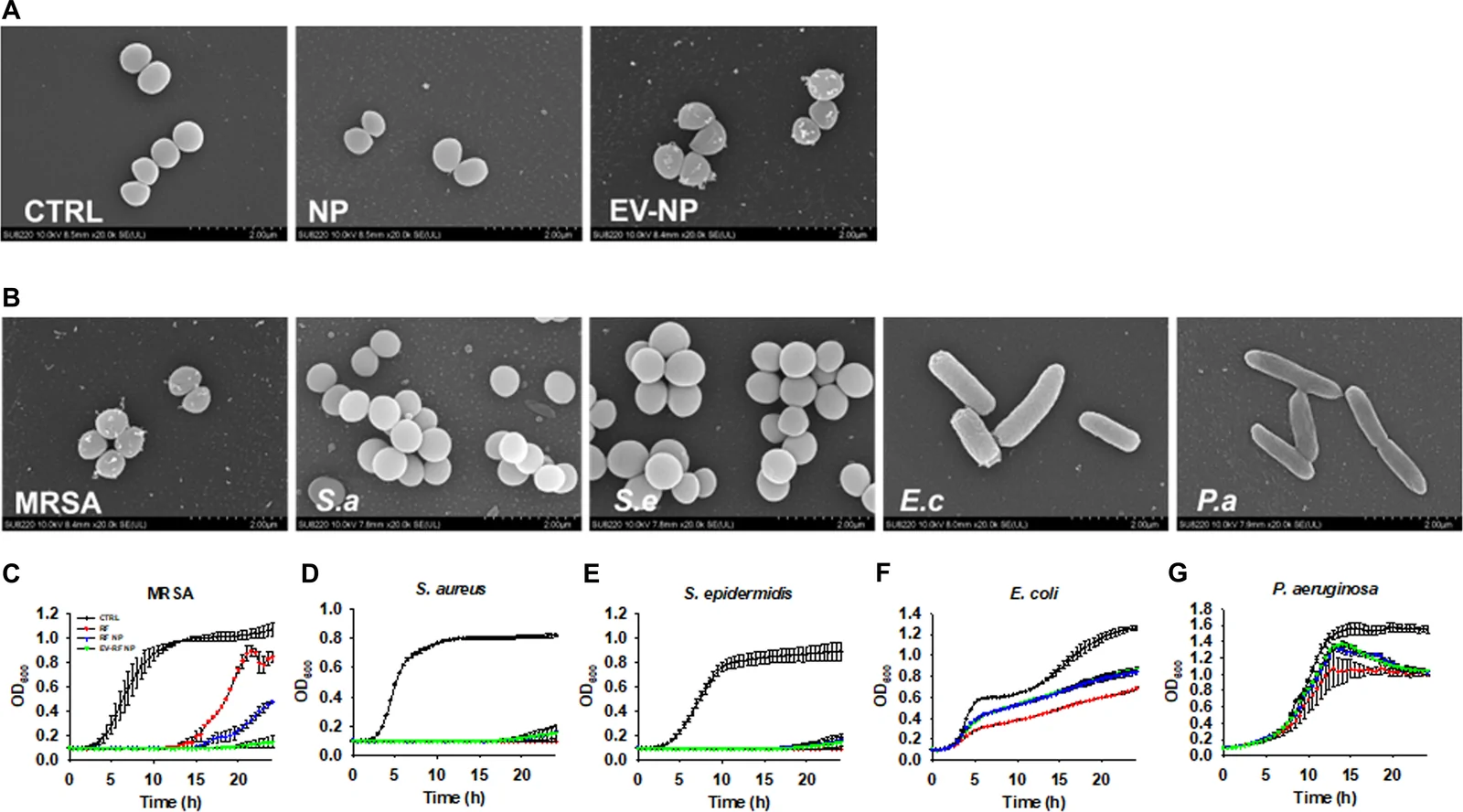
Adhesion of nanocarriers with MRSA and its impact on antibacterial performance. (A) SEM images of the surface interaction between MRSA and NP or EV-NP, showing strong bacterial adhesion exclusively in the EV-NP group. (B) SEM analysis of EV-NP binding to different bacterial species (MRSA, *S. aureus*, *S. epidermidis*, *E. coli*, *P. aeruginosa*), demonstrating selective adhesion only to MRSA. (C to G) Time growth curves evaluating the antibacterial effects of RF, RF NP, and EV-RF NP against MRSA (C), *S. aureus* (D), *S. epidermidis* (E), *E. coli* (F), and *P. aeruginosa* (G). Data are presented as mean ± SEM (*n* = 3).

### EV-coated nanocarrier vaccination protects mice against MRSA infection

The prophylactic efficacy of EV-NP was evaluated in a murine bacteremia model. Mice received subcutaneous EV-NP vaccinations on days −14 and −7, followed by intravenous MRSA challenge on day 0 (Fig. 7A). At 24 h post-infection, MRSA colonization was established in peripheral organs (Fig. 7B). Both EV and EV-NP significantly reduced bacterial burden by 3- to 4-log in the liver, spleen, lung, and kidney. In heart and blood, EV-NP achieved 4- to 5-log reductions, outperforming EV.

**Fig. 7.**
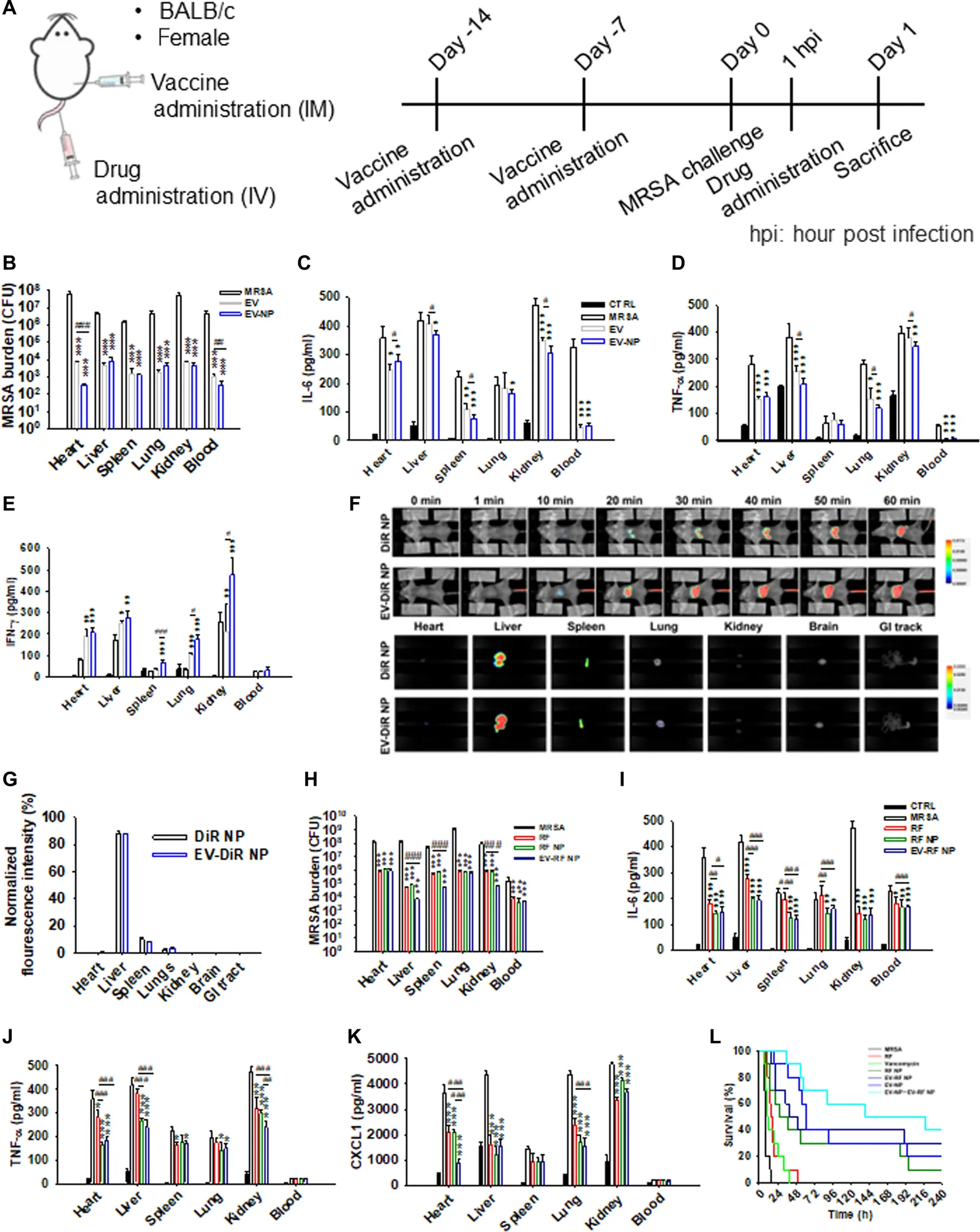
Protective efficacy of the EV-NP vaccine platform and therapeutic performance of EV-RF NP in MRSA-induced bacteremia and their combined impact on mouse survival. (A) Experimental timeline illustrating vaccination, MRSA challenge, and treatment schedules. (B) MRSA burden in major organs from vaccinated mice (MRSA, EV, EV-NP). (C to E) Cytokine levels in vaccinated mice, including IL-6 (C), TNF-α (D), and IFN-γ (E). (F and G) In vivo biodistribution images (F) of DiR-labeled NP and EV-NP nanocarriers 0 to 60 min post-injection and corresponding organ level quantification (G). (H) MRSA burden in major organs following therapeutic treatment (MRSA, RF, RF NP, EV-RF NP). (I to K) Cytokine levels in therapeutically treated mice, including IL-6 (I), TNF-α (J), and CXCL1 (K). (L) Survival analysis of MRSA-infected mice across all treatment groups (*n* = 10). Compared with the untreated MRSA group: *P < 0.05, **P < 0.01, ***P < 0.001. EV versus EV-NP, RF versus RF NP versus EV-RF NP: *P* < 0.05, ^##^*P* < 0.01, ^###^*P* < 0.001.

Proinflammatory cytokines IL-6 and TNF-α were markedly up-regulated upon MRSA infection. EV-NP vaccination more effectively suppressed IL-6 in the liver, spleen, and kidney, while EV failed to reduce IL-6 in the liver and lung (Fig. 7C). Similarly, EV-NP induced stronger TNF-α inhibition in the liver, lung, and kidney (Fig. 7D). MRSA infection also increased interferon-γ (IFN-γ) levels (Fig. 7E). Both EV and EV-NP further elevated IFN-γ, with EV-NP inducing approximately 2-fold higher expression in spleen, lung, and kidney, suggesting enhanced Th1 immune activation.

### EV-coated nanocarriers loaded with RF mitigate bacteremia in mice

To assess therapeutic efficacy, RF-loaded nanoplatforms were administered intravenously 1 h post-MRSA infection. Biodistribution was evaluated using in vivo and ex vivo imaging. Live imaging revealed preferential accumulation of both NP and EV-NP in the liver and spleen, with EV-NP showing faster and stronger hepatic uptake (Fig. 7F, upper panel). Ex vivo imaging at 1 h confirmed this distribution, with minimal signal in kidney, brain, and gastrointestinal (GI) tract (Fig. 7F, lower panel). Quantification showed about 90% of the injected dose localized in the liver (Fig. 7G).

Treatment with free RF, RF NP, and EV-RF NP all reduced MRSA burden in organs and blood (Fig. 7H), with EV-RF NP showing the most pronounced effect. MRSA infection elevated IL-6, TNF-α, and CXCL1 in multiple organs (Fig. 7I to K). Free RF attenuated IL-6 levels, while NP formulations provided further suppression. IL-6 inhibition by RF NP and EV-RF NP was comparable (Fig. 7I). A similar pattern was seen for TNF-α, with EV-RF NP providing superior suppression in the kidney (Fig. 7J). For CXCL1, RF NP and EV-RF NP reduced levels by 2- and 4-fold, respectively (Fig. 7K), although their effects were similar across most organs.

We next assessed survival outcomes in bacteremia mice receiving various treatments: free RF, vancomycin, RF NP, EV-RF NP, EV-NP vaccination, and combined vaccination plus EV-RF NP therapy. Survival was tracked over 10 d. All untreated infected mice died within 24 h (Fig. 7L). Free RF and vancomycin modestly extended survival to longer time. NP-based therapies showed superior protection. EV-NP vaccination and EV-RF NP therapy yielded 20% and 30% survival, respectively. Combined vaccination and treatment further increased survival to 40%. Median survival times were 9 h for control, 17 h for free RF, and 12 h for vancomycin (Table S2). RF NP and EV-RF NP extended median survival to 27 and 41 h, respectively. Notably, the combination strategy prolonged survival to 140 h, demonstrating a synergistic therapeutic benefit.

Hematoxylin and eosin (H&E) histological analysis of peripheral organs revealed differential pathological changes following bacteremia. Despite marked elevations in proinflammatory mediators in the heart, liver, and spleen, no overt structural damage was observed in these organs (Fig. 8A to C). In this experiment, mice were challenged with 1 × 10^8^ CFU/ml MRSA that was 10-fold lower than the dose used in the survival study, likely insufficient to induce severe injury in these tissues. In contrast, significant morphological alterations were apparent in the lung and kidney after MRSA infection (Fig. 8D and E). Infected lungs exhibited interstitial congestion and disrupted pulmonary architecture, accompanied by extensive immune cell infiltration. Free RF moderately attenuated alveolar wall swelling and congestion, while RF delivered by NPs provided further improvement. MRSA infection also induced interstitial edema, congestion, and immune cell aggregates in the kidney. Free RF partially reduced these pathological features, and nanoformulations enhanced renal protection, further mitigating infection-associated injury.

**Fig. 8.**
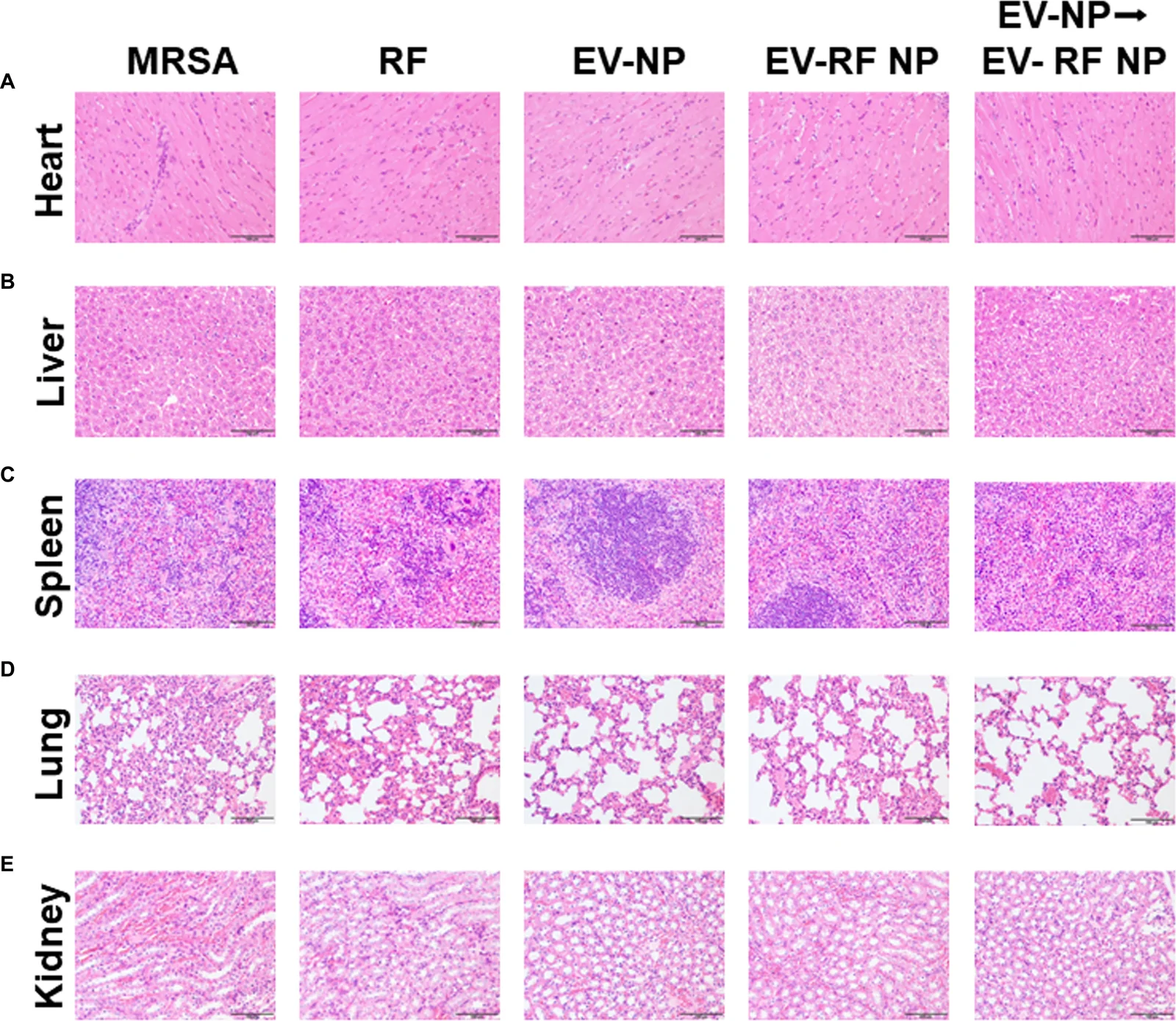
The H&E staining of (A) heart, (B) liver, (C) spleen, (D) lungs, and (E) kidneys in the MRSA-infected mice.

### The nanoplatforms cause no in vivo acute toxicity

The safety of the nanoformulations was assessed following subcutaneous EV-NP vaccination and/or intravenous EV-RF NP administration. All treated mice exhibited 100% survival. H&E staining of major organs at 24 h post-treatment revealed no histopathological abnormalities compared to untreated controls, indicating the absence of acute toxicity (Fig. S7A). Blood biochemistry analysis showed minimal differences in liver and kidney function parameters across all treatment groups relative to healthy controls (Fig. S7B to E).

## Discussion/Conclusion

Bacteria-mimetic therapies offer a promising avenue for infectious disease treatment by leveraging microbial interactions with host immunity [18]. Here, we developed MRSA-derived EV-coated PLGA NPs that function both as immunogenic vaccines and targeted antibiotic carriers. Bacterial EVs are inherently immunomodulatory and serve as multi-antigenic platforms for combating AMR. Our EV-NP system enhanced prophylactic efficacy by stimulating immune responses and, when loaded with RF, simultaneously enabled targeted antimicrobial delivery. These nanocarriers adhered efficiently to planktonic and biofilm-associated MRSA, facilitating bacterial clearance. In a challenging bacteremia model, the dual application of EV-based vaccination and EV-RF NP therapy achieved robust pathogen control. This integrated strategy presents a potent and clinically translatable solution for managing MRSA-induced sepsis, where effective therapies remain limited.

Bacterial EVs facilitate host–pathogen communication by delivering bioactive molecules such as proteins, lipids, and nucleic acids [7], making them promising vaccine vectors. Effective vaccination involves innate immune activation, primarily through APCs like macrophages and dendritic cells, which bridge to adaptive immunity. Our results showed that EV-NP significantly enhanced dendritic cell maturation markers and proinflammatory cytokines, outperforming EV alone. EV-NP also activated macrophages, highlighting the immunogenic potential of bacterial EV. Unlike traditional subunit vaccines that lack antigen diversity, bacterial EVs carry multiple native antigens [19]. Proteomic analysis revealed high levels of autolysin and penicillin-binding protein 2 (PBP2), both involved in MRSA cell wall remodeling and potential antigen recognition [20,21]. Autolysin is a key membrane antigen that triggers innate immunity [22], while PBP2 is central to β-lactam resistance and an attractive vaccine target [23]. Abundant hemolysins in EV-NP may promote antibody production and antivirulence immunity [24]. Additional proteins such as IsaB, LUKLs, and lipases could further contribute to antigenicity [25,26]. Although EV-NP showed potent immunostimulatory effects, the precise antigens driving the protective response require further investigation.

Antigen delivery to APCs relies on both ligand–receptor interactions and intracellular uptake [8], yet achieving efficient antigen presentation remains a challenge. NPs cloaked with bacterial membranes enhance phagocytic uptake by innate immune cells [18]. Consistent with previous findings [27], our study showed that EV-NP promoted significantly higher macrophage internalization and cytokine expression than bare NPs. This improved uptake may result from EV-associated components such as surface glycoproteins and staphylococcal toxins (e.g., hemolysins and LUKLs), which enhance membrane permeability and facilitate phagocytosis via receptors like scavenger, complement, and toll-like receptors [28–30]. In dendritic cells, EV coating also improved NP uptake and up-regulated activation markers, highlighting the potential of EV-NP to function as both antigen carriers and immune adjuvants [31]. Furthermore, EV-NP localized to lysosomes post-internalization, suggesting intracellular degradation of the nanocarrier—a process essential for effective antigen processing and presentation.

The enhanced internalization of EV-NP over native EV led to stronger cytokine release and APC maturation. This process is driven by pathogen-associated molecular patterns (PAMPs), such as EV-derived lipoproteins, which are recognized by pattern recognition receptors (PRRs) [32]. APC maturation is essential for bridging innate and adaptive immunity. Mature APCs activate T and B cells, which mediate cellular cytotoxicity and antibody production, respectively. In vivo, EV-NP triggered both T and B cell responses, as evidenced by increased CD69 expression and elevated IgG titers, indicating robust adaptive immune activation. While bacterial EVs show promise as vaccines, their native form often carries virulence factors that raise toxicity concerns [33]. In our study, EV induced hemolysis, but this effect was fully abolished upon NP coating. Since we just employed EV membrane to be coated on NP surface, some toxic cargos inside EV were absent in EV-NP [34]. Additionally, the nanocarrier matrix may bind and neutralize residual bacterial toxins [35]. Although EV-NP retained the membrane-derived targeting property of EV, its hemolytic activity is substantially reduced compared with that of intact EV.

Although vaccines can elicit potent antibacterial immunity, combining immunization with antibiotic therapy may be necessary to fully eradicate infection. To this end, RF was incorporated into the EV-camouflaged nanoplatform to target MRSA post-vaccination. EV-RF NP markedly inhibited planktonic MRSA growth and lowered the MIC relative to free RF. In contrast, free RF at sub-MIC doses (25 to 50 ng/ml) initially suppressed growth but later allowed bacterial regrowth, consistent with the phenomenon that sub-inhibitory antibiotic exposure can promote resistance and rebound proliferation [36,37].

The superior late-stage activity of EV-RF NP is unlikely to result from direct NP internalization, as bacteria generally do not internalize particles >20 nm [38]. Instead, effective antibacterial delivery required close association between the nanocarrier and the bacterial surface. EVs preferentially bind to homologous bacteria by mimicking native cell surface characteristics [39]. Our SEM confirmed that EV-NP but not bare NPs adhered selectively to MRSA. This specific homotypic adhesion enhances local RF concentration at the bacterial envelope, facilitating penetration and activity. A similar targeting effect has been observed with RF-loaded NPs coated with *E. coli* EV against *E. coli* but not *S. aureus* [40]. Another explanation is that methicillin resistance is associated with substantial alterations in the cell envelop architecture of MRSA, including changes in wall teichoic acid composition, surface glycosylation, and membrane-associated proteins, which differ from those of methicillin-susceptible *S. aureus* (MSSA) [41,42]. Since EV-NP was derived from MRSA EV, these shared surface characteristics may promote preferential homotypic interaction with MRSA than MSSA. We hypothesize that the attachment of EV-RF NP on MRSA surface could increase local accumulation of RF near the bacteria. Subsequently PLGA degraded to release RF molecules for passive penetration into bacteria. Moreover, EV-NP enriched in pore-forming components (e.g., autolysin, hemolysins, and leukotoxins) may compromise the MRSA envelope, thereby enabling antibiotic entry into the intracellular space and improving bactericidal efficacy.

In the case of MSSA and *S. epidermidis*, both free and nanoencapsulated RF exhibited comparable antibacterial activities, resulting in nearly complete bacterial eradication. Unlike MRSA, the enhanced antibacterial effect of EV-RF NP against these strains was not associated with preferential EV-mediated attachment, suggesting that bacterial killing was primarily mediated by the release of RF from the nanocarriers. These findings indicate that the proposed nanoplatform is applicable to both drug-resistant and drug-sensitive bacteria. Nevertheless, its greatest therapeutic advantage is expected for MRSA infections, where conventional antibiotic therapy is often compromised by AMR. In contrast, MSSA infections generally remain susceptible to standard antibiotic treatment, reducing the added clinical benefit of a more sophisticated nanocarrier-based delivery system.

MRSA bacteremia remains challenging to treat due to bacterial survival and replication within phagocytic cells, particularly macrophages and neutrophils [27]. Effective clearance of intracellular MRSA is therefore essential. However, RF exhibits limited cellular penetration, impairing its ability to eliminate intracellular pathogens [43]. In this study, EV-NP encapsulation significantly enhanced RF-mediated intracellular bacterial clearance in both macrophages and neutrophils. This improvement may stem from the membrane-associated proteins on MRSA-derived EVs, which facilitate mimicry of bacterial surface features and promote host–pathogen interactions. EV-RF NP, resembling native MRSA, could be more efficiently internalized by APCs through phagocytosis. Upon re-exposure, APCs rapidly recognize PAMPs, enabling rapid uptake. This “Trojan horse” strategy allowed RF to enter infected phagocytes and exert intracellular antibacterial activity. Neutrophils also internalized EV-RF NP, likely via PAMP recognition by toll-like and scavenger receptors [44]. The presence of these PAMPs on EV-NP surfaces contributed to enhanced ligand–receptor binding and cellular uptake, facilitating intracellular MRSA elimination.

MRSA aggregates form dense biofilms that hinder antibiotic penetration, contributing significantly to AMR in bacteremia treatment [13]. Although RF has documented anti-biofilm activity [45], our data showed that free RF reduced viable MRSA within mature biofilms but failed to disrupt the biomass, as indicated by crystal violet staining. This suggests that RF penetrated the biofilm to kill bacteria without affecting the extracellular matrix. Biofilm penetration remains a major challenge, especially in mature structures [46]. Confocal imaging with SYTO9 revealed that free RF primarily eradicated bacteria in the superficial layers. In contrast, RF-loaded NPs not only inhibited bacterial growth but also disrupted the biofilm structure, likely due to enhanced penetration and wider distribution within the matrix. This superior performance may be attributed to the biomimetic features of EV-RF NP, which facilitate coaggregation with MRSA and adherence to biofilms [47]. EV-associated proteins such as autolysin and thermonuclease, known to degrade extracellular DNA that is a key biofilm component, likely contributed to the enhanced biomass breakdown [48,49].

Bacteremia-induced sepsis is a life-threatening condition marked by cytokine storm and multiorgan failure, particularly affecting the heart, liver, lungs, and kidneys [50]. Our in vivo data demonstrated that both EV-NP vaccination and EV-RF NP therapy significantly reduced MRSA burden in circulation and major organs, outperforming free RF and plain NPs. This bacterial clearance correlated with reduced proinflammatory cytokine levels and improved survival outcomes. Although vancomycin is a frontline treatment for bacteremia, its limited efficacy here may be attributed to subtherapeutic dosing (1 μg) and strain resistance. Notably, myocardial injury is linked to high mortality in sepsis. EV-NP conferred robust cardiac protection, markedly reducing MRSA load and CXCL1 levels in the heart. Enhanced accumulation of EV-NP in the heart and liver suggests improved targeting and prolonged in vivo residence, potentially sustaining immune activation and therapeutic effect.

Despite the kidney’s filtration cutoff (about 6 nm) limiting NP retention, EV-NP still effectively reduced renal MRSA burden and cytokine levels, likely due to upstream bacterial suppression in circulation. EV-NP also induced higher IFN-γ secretion than EV, supporting the development of long-lasting adaptive immunity through Th1 activation. These findings reinforce the value of vaccine-driven prevention against AMR pathogens, preserving antibiotic efficacy [11]. The combined prophylactic and therapeutic strategy using EV-based nanoplatforms yielded synergistic benefits, significantly extending survival.

A major challenge for bacteria-based vaccines and therapeutics is achieving an optimal balance between immunogenicity and safety [18]. Bacterial EV can carry virulence factors that trigger toxicity and excessive inflammation; for example, circulating *S. aureus* EVs have been associated with hemolysis [8,21]. In contrast, our safety evaluations including hemolysis assays, cell viability, and serum biochemistry demonstrated that EV-coated nanocarriers are well tolerated. A key advantage of EV-NP over native EV is its improved safety profile, providing a more translatable platform without compromising immunogenicity. Effective management of MRSA bacteremia requires both rapid control of circulating bacteria and clearance of pathogens within peripheral organs. To summarize the overarching strategy of this work, we propose a dual-functional EV-based nanoplatform that integrates vaccination and antibiotic therapy (Fig. 9).

**Fig. 9.**
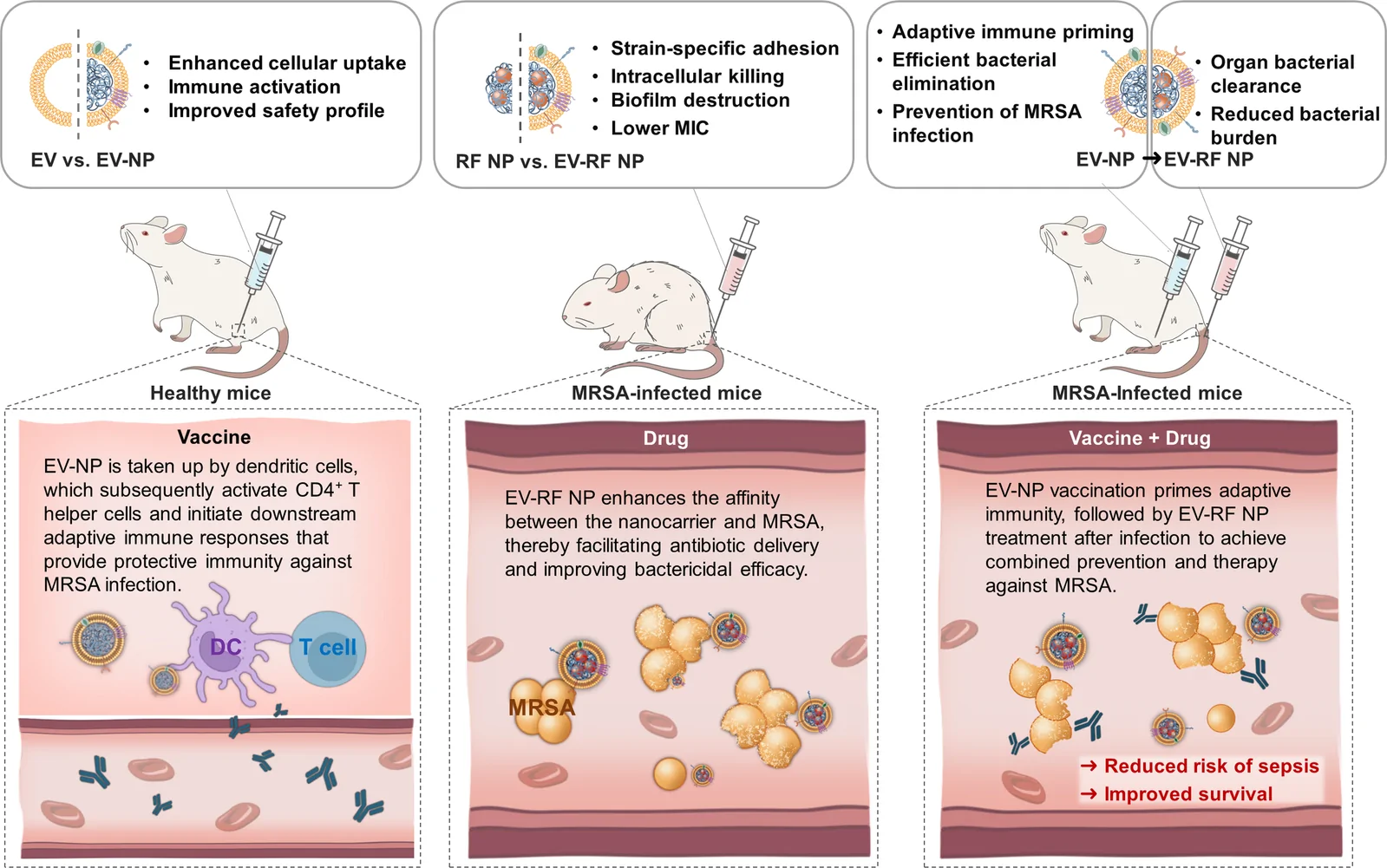
The illustration of the mechanisms of the EV-NP nanoplatforms for combined prophylactic and therapeutic treatments against MRSA bacteremia.

This study has some limitations. The bacteremia pathogenesis in animal models does not fully recapitulate that in humans, limiting direct clinical translatability. The short duration of in vivo experiments also precluded assessment of long-term outcomes. In addition, our EV-NP was derived from a specific MRSA strain. Its generalizability to other clinical isolates remains uncertain. Challenges such as scalability, batch-to-batch variability, and regulatory hurdles continue to impede the clinical translation of EV-based therapeutics. Further optimization and standardization are essential for advancing these nanoplatforms toward clinical use.

We successfully developed MRSA EV-based biomimetic nanocarriers capable of both immunization and targeted antimicrobial therapy. By mimicking the multi-antigenic profile of native EVs, the nanoplatform promoted dendritic cell maturation, T and B cell activation, and antibody production. When loaded with RF, the system achieved selective adhesion to MRSA, enabling efficient drug delivery across host cell membranes and biofilms. In vivo, these nanoformulations significantly reduced bacterial burden and inflammatory responses in a bacteremia model. Given the repeated clinical failures of *S. aureus* vaccines, this strategy represents a promising alternative that unites immune priming with pathogen-specific antibacterial action to address multidrug-resistant infections.

## Data Availability

Data are available on request from the authors.
