# Supplementary material for: Extracellular Vesicle-Mimetic Nanoplatforms for Integrated Vaccination and Targeted Antibiotic Therapy against MRSA Bacteremia: A Wolf in Sheep’s Clothing
Source: Biomater Res. 2026 Sep 30;30:0411. doi: 10.34133/bmr.0411 (PMC13624486; doi:10.34133/bmr.0411)
Supplement: Supplementary file 1 [file bmr.0411.f1.zip › Supplementary Figures.pdf]

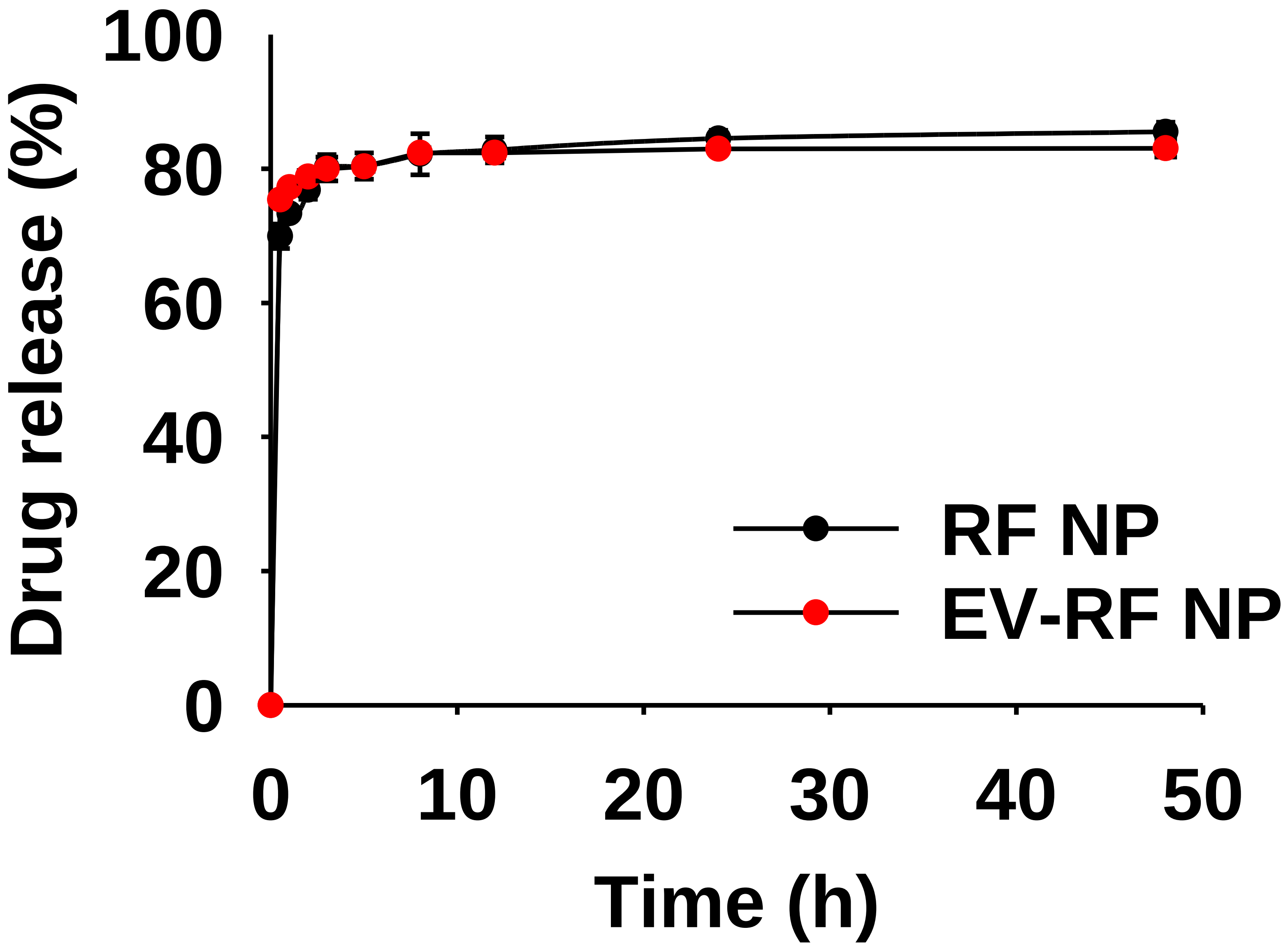

Suppl. Fig. 1

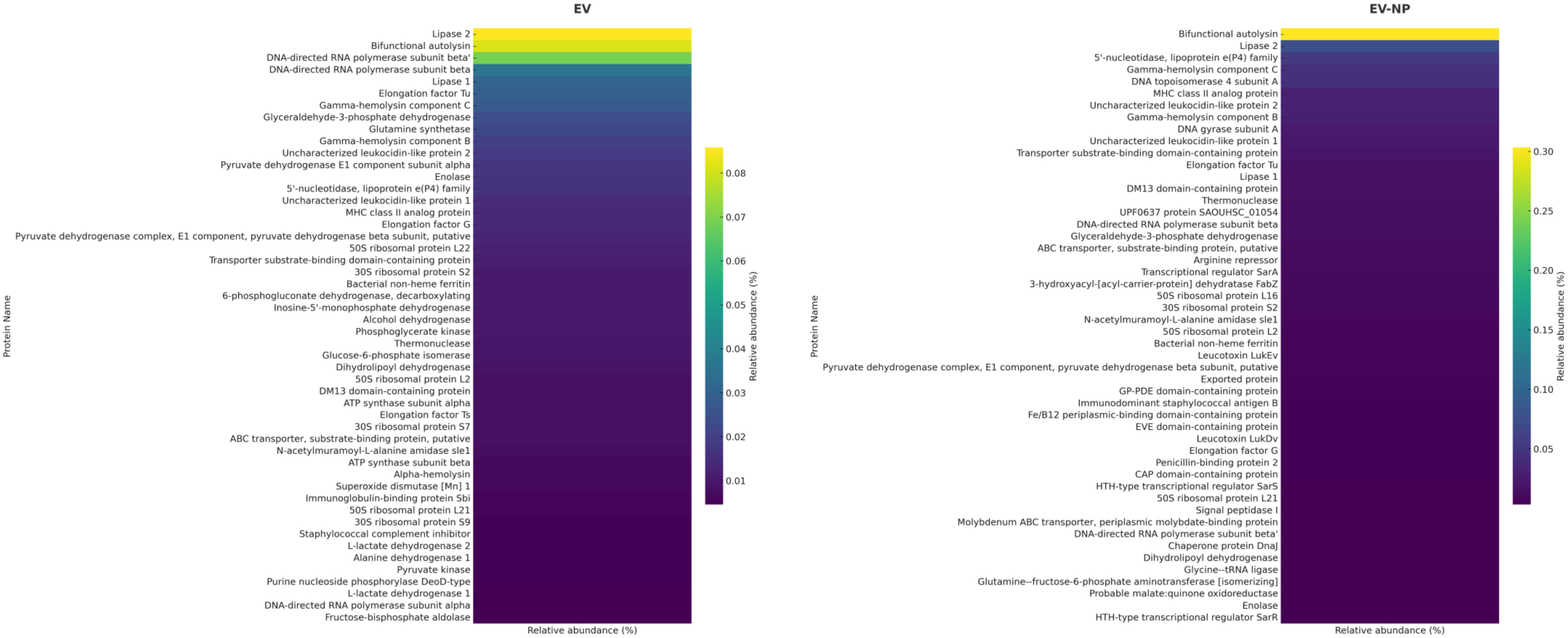
EV
EV-NP
Protein Name
Relative abundance (%)
Lipase 2
Bifunctional autolysin
DNA-directed RNA polymerase subunit beta'
DNA-directed RNA polymerase subunit beta
Lipase 1
Elongation factor Tu
Gamma-hemolysin component C
Glyceraldehyde-3-phosphate dehydrogenase
Glutamine synthetase
Gamma-hemolysin component B
Uncharacterized leukocidin-like protein 2
Pyruvate dehydrogenase E1 component subunit alpha
Enolase
5'-nucleotidase, lipoprotein e(P4) family
Uncharacterized leukocidin-like protein 1
MHC class II analog protein
Elongation factor G
Pyruvate dehydrogenase complex, E1 component, pyruvate dehydrogenase beta subunit, putative
50S ribosomal protein L22
Transporter substrate-binding domain-containing protein
30S ribosomal protein S2
Bacterial non-heme ferritin
6-phosphogluconate dehydrogenase, decarboxylating
Inosine-5'-monophosphate dehydrogenase
Alcohol dehydrogenase
Phosphoglycerate kinase
Thermonuclease
Glucose-6-phosphate isomerase
Dihydrolipoyl dehydrogenase
50S ribosomal protein L2
DM13 domain-containing protein
ATP synthase subunit alpha
Elongation factor Ts
30S ribosomal protein S7
ABC transporter, substrate-binding protein, putative
N-acetylmuramoyl-L-alanine amidase sle1
ATP synthase subunit beta
Alpha-hemolysin
Superoxide dismutase [Mn] 1
Immunoglobulin-binding protein Sbi
50S ribosomal protein L21
30S ribosomal protein S9
Staphylococcal complement inhibitor
L-lactate dehydrogenase 2
Alanine dehydrogenase 1
Pyruvate kinase
Purine nucleoside phosphorylase DeoD-type
L-lactate dehydrogenase 1
DNA-directed RNA polymerase subunit alpha
Fructose-bisphosphate aldolase
0.08
0.07
0.06
0.05
0.04
0.03
0.02
0.01
Bifunctional autolysin
Lipase 2
5'-nucleotidase, lipoprotein e(P4) family
Gamma-hemolysin component C
DNA topoisomerase 4 subunit A
MHC class II analog protein
Uncharacterized leukocidin-like protein 2
Gamma-hemolysin component B
DNA gyrase subunit A
Uncharacterized leukocidin-like protein 1
Transporter substrate-binding domain-containing protein
Elongation factor Tu
Lipase 1
DM13 domain-containing protein
Thermonuclease
UPF0637 protein SAOUHSC_01054
DNA-directed RNA polymerase subunit beta
Glyceraldehyde-3-phosphate dehydrogenase
ABC transporter, substrate-binding protein, putative
Arginine repressor
Transcriptional regulator SarA
3-hydroxyacyl-[acyl-carrier-protein] dehydratase FabZ
50S ribosomal protein L16
30S ribosomal protein S2
N-acetylmuramoyl-L-alanine amidase sle1
50S ribosomal protein L2
Bacterial non-heme ferritin
Leucotoxin LukEv
Pyruvate dehydrogenase complex, E1 component, pyruvate dehydrogenase beta subunit, putative
Exported protein
GP-PDE domain-containing protein
Immunodominant staphylococcal antigen B
Fe/B12 periplasmic-binding domain-containing protein
EVE domain-containing protein
Leucotoxin LukDv
Elongation factor G
Penicillin-binding protein 2
CAP domain-containing protein
HTH-type transcriptional regulator SarS
50S ribosomal protein L21
Signal peptidase I
Molybdenum ABC transporter, periplasmic molybdate-binding protein
DNA-directed RNA polymerase subunit beta'
Chaperone protein DnaJ
Dihydrolipoyl dehydrogenase
Glycine--tRNA ligase
Glutamine--fructose-6-phosphate aminotransferase [isomerizing]
Probable malate:quinone oxidoreductase
Enolase
HTH-type transcriptional regulator SarR
0.30
0.25
0.20
0.15
0.10
0.05

Suppl. Fig. 2

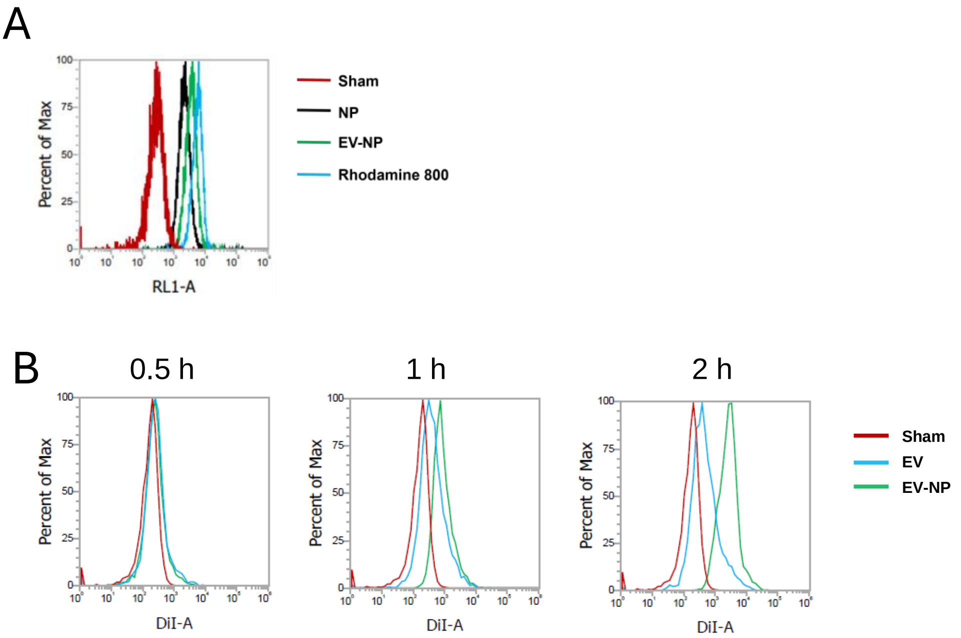
A
Percent of Max
RL1-A
Sham
NP
EV-NP
Rhodamine 800
B
0.5 h
1 h
2 h
Percent of Max
DiI-A
Sham
EV
EV-NP

Suppl. Fig. 3

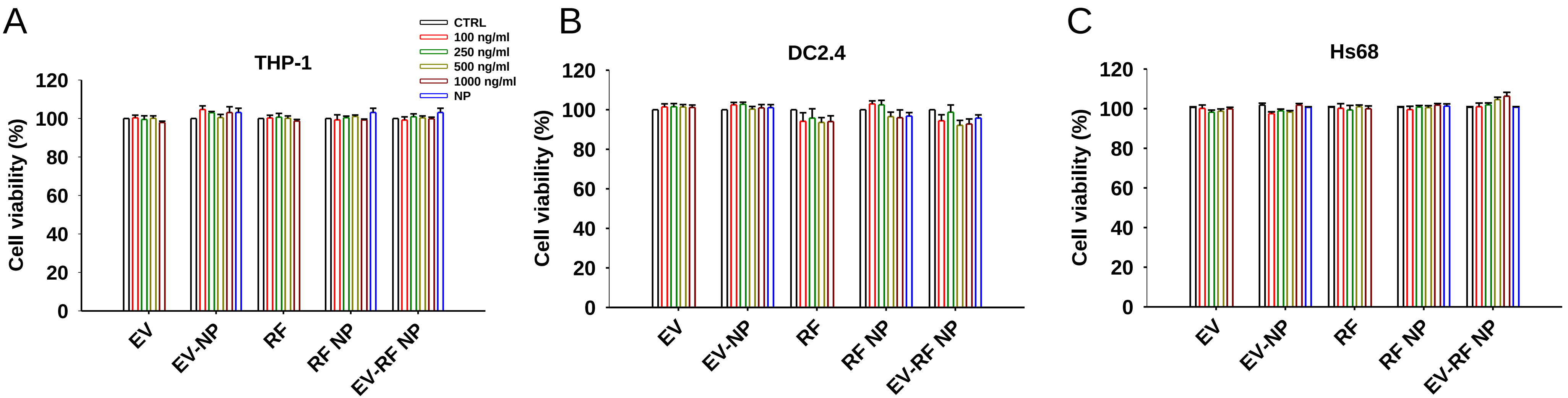

A
THP-1
CTRL
100 ng/ml
250 ng/ml
500 ng/ml
1000 ng/ml
NP
Cell viability (%)
0
20
40
60
80
100
120
EV
EV-NP
RF
RF NP
EV-RF NP
B
DC2.4
Cell viability (%)
0
20
40
60
80
100
120
EV
EV-NP
RF
RF NP
EV-RF NP
C
Hs68
Cell viability (%)
0
20
40
60
80
100
120
EV
EV-NP
RF
RF NP
EV-RF NP

Suppl. Fig. 4

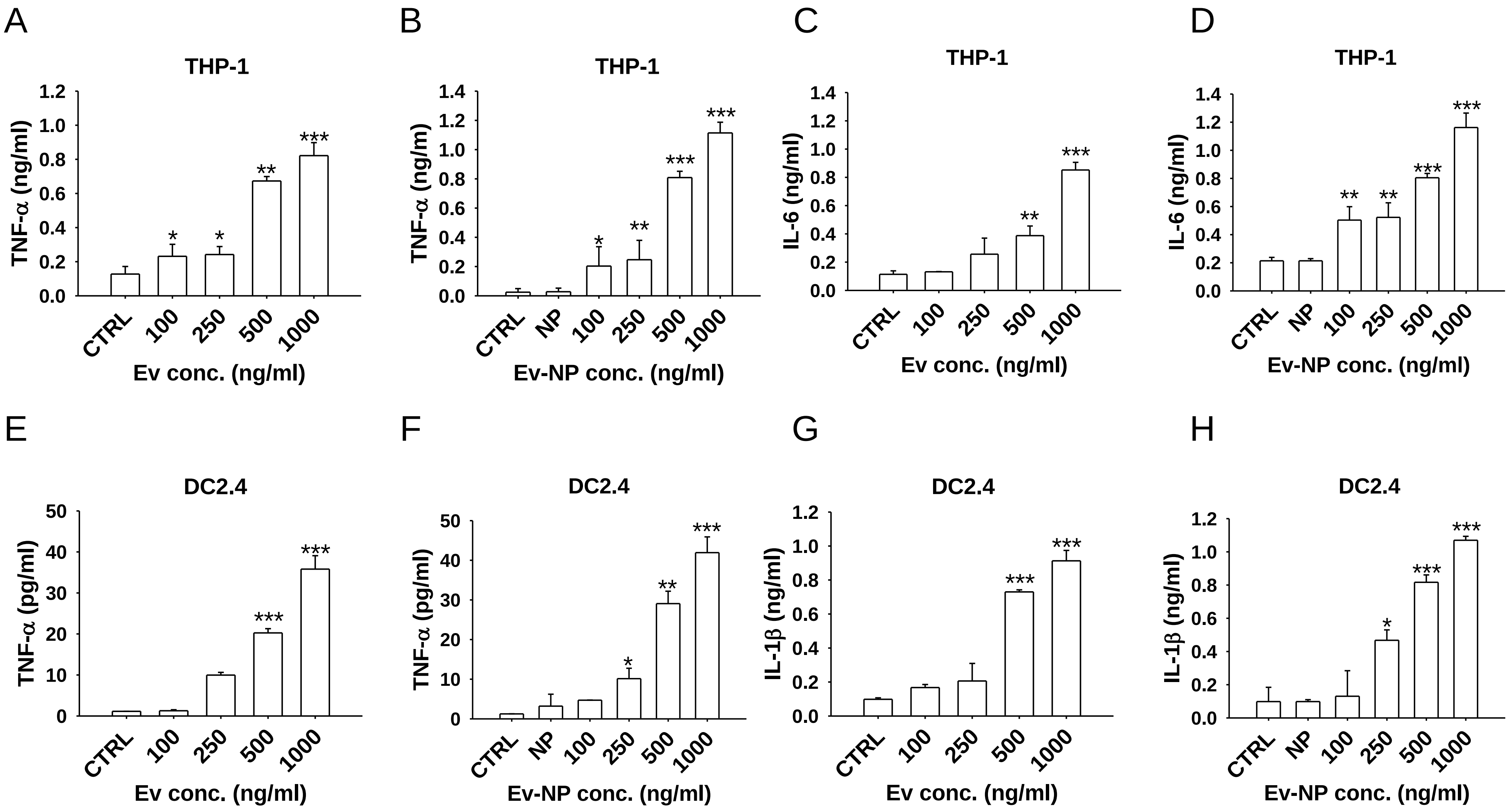
A
THP-1
TNF-α (ng/ml)
Ev conc. (ng/ml)
B
THP-1
TNF-α (ng/m)
Ev-NP conc. (ng/ml)
C
THP-1
IL-6 (ng/ml)
Ev conc. (ng/ml)
D
THP-1
IL-6 (ng/ml)
Ev-NP conc. (ng/ml)
E
DC2.4
TNF-α (pg/ml)
Ev conc. (ng/ml)
F
DC2.4
TNF-α (pg/ml)
Ev-NP conc. (ng/ml)
G
DC2.4
IL-1β (ng/ml)
Ev conc. (ng/ml)
H
DC2.4
IL-1β (ng/ml)
Ev-NP conc. (ng/ml)
CTRL
NP
100
250
500
1000

Suppl. Fig. 5

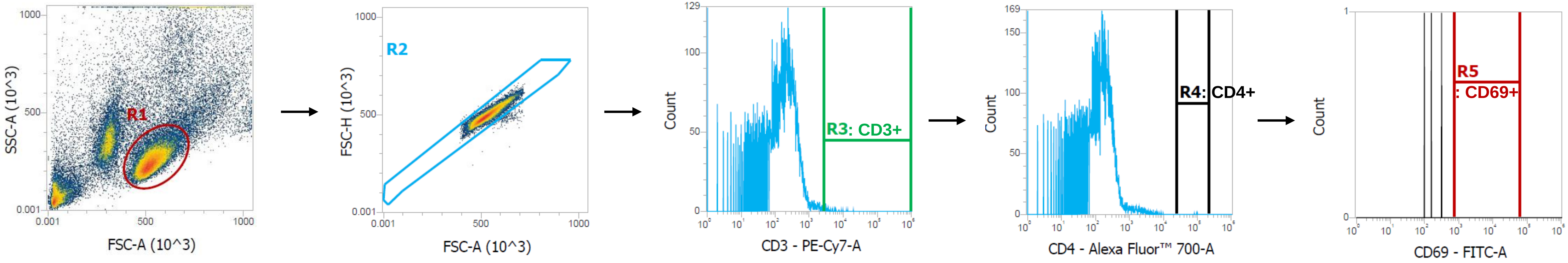

SSC-A (10^3)
FSC-A (10^3)
R1
FSC-H (10^3)
R2
Count
CD3 - PE-Cy7-A
R3: CD3+
CD4 - Alexa Fluor™ 700-A
R4: CD4+
CD69 - FITC-A
R5
: CD69+

Suppl. Fig. 6

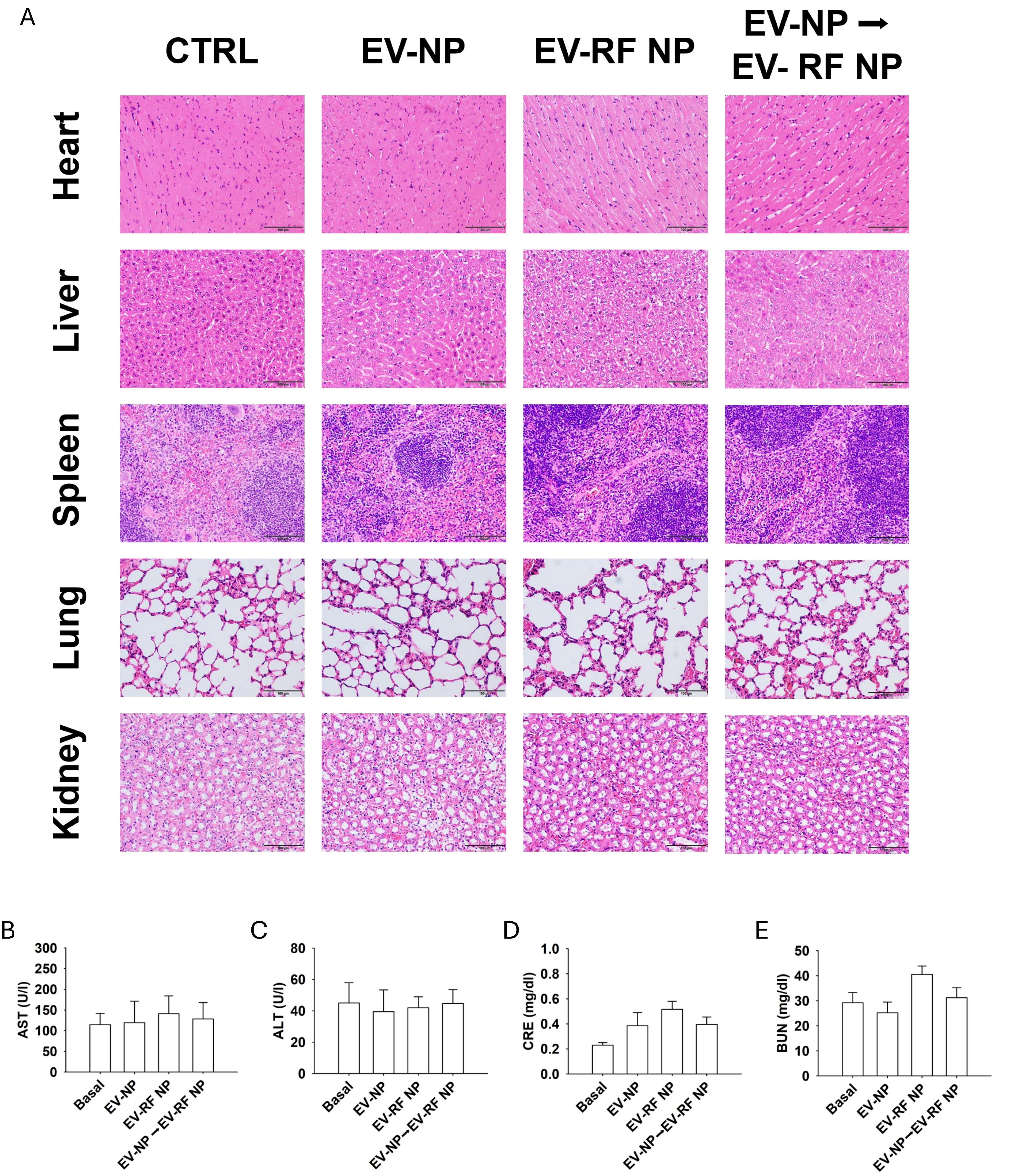
A
CTRL
EV-NP
EV-RF NP
EV-NP → EV- RF NP
Heart
Liver
Spleen
Lung
Kidney
B
AST (U/l)
300
250
200
150
100
50
0
Basal
EV-NP
EV-RF NP
EV-NP→EV-RF NP
C
ALT (U/l)
80
60
40
20
0
Basal
EV-NP
EV-RF NP
EV-NP→EV-RF NP
D
CRE (mg/dl)
1.0
0.8
0.6
0.4
0.2
0.0
Basal
EV-NP
EV-RF NP
EV-NP→EV-RF NP
E
BUN (mg/dl)
50
40
30
20
10
0
Basal
EV-NP
EV-RF NP
EV-NP→EV-RF NP

Suppl. Fig. 7
