# Supplementary material for: Extracellular Vesicle-Mimetic Nanoplatforms for Integrated Vaccination and Targeted Antibiotic Therapy against MRSA Bacteremia: A Wolf in Sheep’s Clothing
Source: Biomater Res. 2026 Sep 30;30:0411. doi: 10.34133/bmr.0411 (PMC13624486; doi:10.34133/bmr.0411)
Supplement: Supplementary file 1 [file bmr.0411.f1.zip › Supplementary Materials.docx]

**Supplementary Materials**

Suppl. Figure 1. The release profiles of RF from RF NP and RF-EV NP. Data are presented as mean±S.E.M. (n=3).

Suppl. Figure 2. Heatmap showing the top 50 most abundant proteins identified in native MRSA-derived EV and EV-NP.

Suppl. Figure 3. Cellular uptake of nanocarriers or EV analyzed by flow cytometry: (A) Flow cytometric analysis of THP-1 macrophage-like cells after 1 h incubation with dye-labeled NP or EV-NP, showing enhanced uptake of EV-NP compared with uncoated NP. (B) Time-dependent uptake of DiI-labeled EV and EV-NP by DC2.4 dendritic cells was measured at 0.5, 1, and 2 h, demonstrating superior and more rapid internalization of EV-NP.

Suppl. Figure 4. Cytotoxicity of EV, EV-NP, RF, RF NP, and EV-RF NP in (A) THP-1 cells, (B) DC2.4 dendritic cells, and (C) Hs68 fibroblasts. Cells were treated with the indicated formulations at concentrations corresponding to rifampicin equivalents ranging from 100 to 1000 ng/ml. For EV and EV-NP groups, doses were normalized to match the equivalent rifampicin concentrations. Cell viability was assessed using the CCK-8 assay. Data are presented as mean±S.E.M. (n=3).

Suppl. Figure 5. Immunostimulatory effects of EV and EV-NP on THP-1 and DC2.4 Cells: (A, B) TNF-α production in THP-1 cells after 24 h stimulation with EV (A) or EV-NP (B). (C, D) IL-6 secretion in THP-1 cells after 24 h stimulation with EV (C) or EV-NP (D). (E, F) TNF-α production in DC2.4 cells after 24 h stimulation with EV (E) or EV-NP (F). (G, H) IL-1β secretion in DC2.4 cells after 24 h stimulation with EV (G) or EV-NP (H). Data are presented as mean±S.E.M. (n=3). Compared with the control (CTRL) group: **p* < 0.05, ***p* < 0.01, ****p* < 0.001.

Suppl. Figure 6. Gating strategy for in vivo T cell activation: Cells were gated out from debris on the forward scatter (FSC) and side scatter (SSC) plot. The cells were identified prior to gating for CD3^+^, CD4^+^ T cells, and were analyzed for expression of the activation marker CD69.

Suppl. Figure 7. In vivo safety evaluation of EV-based vaccine and drug-delivery formulations: (A) Histopathological examination of major organs (heart, liver, spleen, lungs, and kidneys) from healthy mice treated with EV-NP, EV-RF NP, or the combined EV-NP→EV-RF NP formulation. H&E staining revealed no observable tissue damage, including no signs of congestion, necrosis, or hemorrhage. (B–E) Serum biochemical analysis of liver and kidney function markers, including aspartate aminotransferase (AST) (B), alanine aminotransferase (ALT) (C), creatinine (CRE) (D), and blood urea nitrogen (BUN) (E). All measured values remained within the normal physiological range and showed no significant differences compared with untreated controls, indicating good biocompatibility of the EV-based nanoformulations. Data are presented as mean±S.E.M. (n=5–6).
