# Supplementary material for: Extracellular Vesicle-Mimetic Nanoplatforms for Integrated Vaccination and Targeted Antibiotic Therapy against MRSA Bacteremia: A Wolf in Sheep’s Clothing
Source: Biomater Res. 2026 Sep 30;30:0411. doi: 10.34133/bmr.0411 (PMC13624486; doi:10.34133/bmr.0411)
Supplement: Supplementary file 1 [file bmr.0411.f1.zip › Suppl. Tables.docx]

Suppl. Table 1. The MIC of different rifampicin formulations

| **Fomulation** | **MIC (ng/ml)** |
| --- | --- |
| RF | 100–200 |
| RF NP | 50–100 |
| EV-RF NP | 25.5–50 |

Data are presented as mean±S.E.M. (n=3).

Suppl. Table 2. Median survival time of MRSA-infected mice following vaccination, antibiotic therapy, or combined treatment

| **Groups** | **Median survival time (h)** |
| --- | --- |
| Non-treatment control | 9 |
| RF | 17 |
| Vancomycin | 12 |
| RF NP | 27 |
| EV-RF NP | 41 |
| EV-NP | 62 |
| EV-NP**⮕**EV-RF NP | 140 |
